# N6-methyladenosine of *Socs1* modulates macrophage inflammatory response in different stiffness environments

**DOI:** 10.7150/ijbs.74196

**Published:** 2022-09-11

**Authors:** Zhekai Hu, Yuqing Li, Weihao Yuan, Lijian Jin, Wai Keung Leung, Chengfei Zhang, Yanqi Yang

**Affiliations:** 1 Division of Paediatric Dentistry and Orthodontics, Faculty of Dentistry, The University of Hong Kong, Hong Kong SAR, China.; 2 Division of Periodontology and Implant Dentistry, Faculty of Dentistry, The University of Hong Kong, Hong Kong SAR, China.; 3 School of Dentistry, University of California, Los Angeles, Los Angeles, CA 90095, USA.; 4 Division of Restorative Dental Sciences, Faculty of Dentistry, The University of Hong Kong, Hong Kong SAR, China.

**Keywords:** m^6^A, FTO, SOCS1, Hydrogel stiffness, inflammatory response

## Abstract

Macrophages exhibit diverse functions within various tissues during the inflammatory response, and the physical properties of tissues also modulate the characteristics of macrophages. However, the underlying N6-methyladenosine (m^6^A)-associated molecular mechanisms remain unclear. Accordingly, we examined the potential role of m^6^A in macrophage activation and stiffness sensing. Intriguingly, we found that the macrophage inflammatory response and global levels of m^6^A were stiffness-dependent and that this was due to mechanically loosening the chromatin and epigenetic modification (H3K36me2 and HDAC3). In addition, we targeted *suppressor of cytokine signalling 1 (Socs1)* m^6^A methylation in a stiffness-dependent manner by screening the sequencing data and found that a higher stiffness hydrogel activated Jak-STAT and NFκB signalling and suppressed Fto gene expression. Next, by using the CRISPR/Cas9 system to knockout the FTO gene in macrophages, we demonstrated that FTO affects the stiffness-controlled macrophage inflammatory response by sustaining the negative feedback generated by SOCS1. Finally, we determined that the m^6^A reader YTHDF1 binds *Socs1* mRNA and thereby maintains expression of SOCS1. Our results suggest that the FTO/*Socs1*/YTHDF1 regulatory axis is vital to the stiffness-controlled macrophage inflammatory response and that the deletion of FTO affects the negative feedback control exerted by SOCS1. Our findings increase understanding of the regulatory mechanisms involved in macrophage activation and the control of inflammation.

## Introduction

Macrophages are a vital component of the immune system as they recognise changes in adjacent microenvironments and modulate their functions in the inflammatory response 1. Microenvironmental changes such as changes in tissue stiffness are sensed via biochemical signals and mechanical cues 2. In addition, different tissues within the body have distinct stiffnesses, which are affected by pathological conditions 3. For instance, inflamed tissue and tumours are often stiffer than healthy tissue 4. Macrophages are mechanosensitive cells and thus have functions modulated by biophysical cues 5; for example, exocellular biophysical cues contribute to the macrophage inflammatory response, and higher stiffness is correlated with pro-inflammatory activation 6, 7. However, despite the discovery of a positive association between the macrophage inflammatory response and tissue stiffness, the underlying molecular alterations and biological mechanisms remain unclear.

The macrophage inflammatory response is dependent on cell shape 8, and thus macrophage responses differ according to spatial conditions: macrophage elongation has a synergistic effect with interleukin (IL)4/IL13, inducing a pro-healing phenotype-associated alteration that is known as M2 polarisation 9. Macrophage spreading and enlargement further activate the pro-inflammatory phenotype, known as M1 polarisation, which is regulated via epigenetic alteration 10: Jain and Vogel showed that spatial confinement compacts chromatin and alters the inflammatory response of macrophage with epigenetic alterations, i.e., the levels of histone deacetylase 3 [HDAC3] and histone H3 lysine 36 dimethylation [H3K36me2] 11. Additionally, Liu et al. demonstrated that changes in stiffness affect the spatial structure and inflammatory response of macrophages; compared with soft extracellular matrix hydrogels, stiff glass and polystyrene increase macrophage spreading and enhance M1 polarisation 6, 12, 13. Therefore, the macrophage inflammatory response to varying stiffnesses is shape-dependent and controlled by epigenetic DNA alterations, such as histone modifications.

Analogously, RNA is affected by a modification system that directly affects the process and efficiency of mRNA translation 14. For example, the reversible methylation of adenosine to form N6-methyladenosine (m^6^A) is one of the most abundant epigenetic modifications in RNA and plays an essential role in modulating downstream protein expression 15. The generation and degradation of m^6^A methylation are catalysed by enzymes, such as writers [methyltransferase-like (METTL)3, METTL14, and WT1-associated protein (WTAP)], and erasers [fat mass and obesity-associated protein (FTO) and AlkB homologs (ALKBH)5]. Additionally, m^6^A can be recognised by the readers YTH N6-methyladenosine RNA binding protein (YTHDF)1, YTHDF2 and YTHDF3 16. Recent studies have demonstrated that inflammation affects the level of m^6^A, and that regulating the associated enzymes can affect the pathogenicity of inflammation 17. Specifically, Jian et al. discovered that METTL14 aggravates endothelial inflammation by enhancing the levels of m^6^A in forkhead box transcription factor (*FOXO*)*1* mRNA 18; Wu et al. showed that m^6^A restrains inflammation by interacting with DNA histone modification 19; and Du et al. reported that METTL14 inhibits acute bacterial infections by exerting negative feedback control of SOCS1 20. However, the biological role of m^6^A in the mechanical macrophage inflammatory response and the molecular basis of its interaction with other epigenetics-associated modulation demand further investigations.

The present study found that higher hydrogel stiffness enhanced macrophage spreading and epigenetic alteration. Hydrogel stiffness was positively correlated with the level of m^6^A during the macrophage inflammatory response and modulated the balance between FTO and m^6^A methylation in *Socs1* mRNA. Moreover, the mechanical microenvironment tuned the macrophage inflammatory response via the inhibition of FTO. Thus, the creation of FTO knockout (KO) macrophages using the clustered regularly interspaced short palindromic repeat (CRISPR)/CRISPR-associated protein 9 (Cas9) system reduced inflammation by enhancing the level of m^6^A in *Socs1* mRNA. Finally, YTHDF1 was determined as the m^6^A reader that recognises m^6^A-rich *Socs1* mRNA and enhances its stability, thereby facilitating its translation.

## Results

### Higher stiffness enhances systemic prevalence of m^6^A and enlarges the cell and nucleus

**Figure 1:**
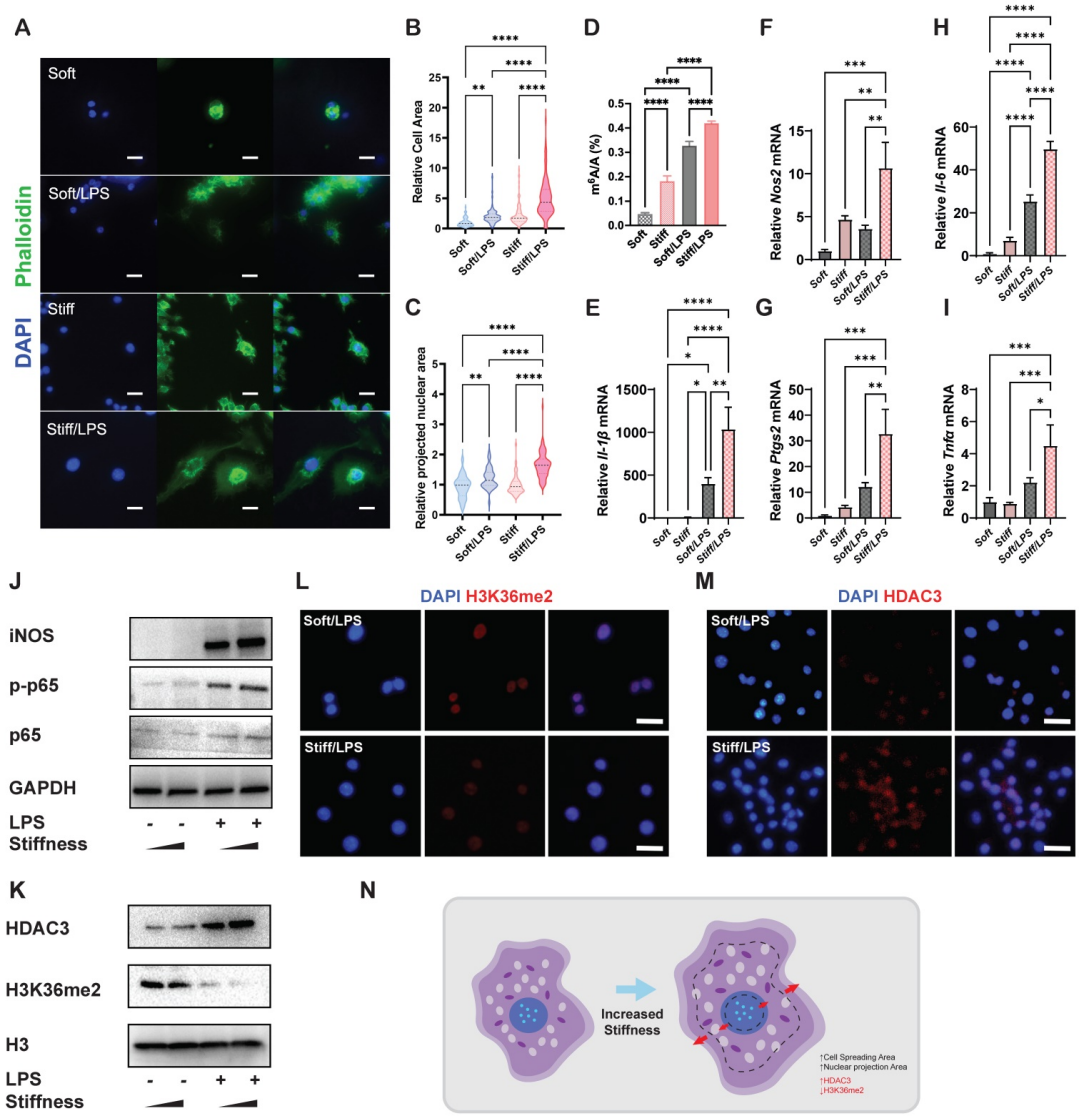
** Stiffness determines the cellular structure-associated macrophage inflammatory response mediated by HDAC3 and H3K36me2. A.** Representative images of macrophages on soft or stiff hydrogel with/without LPS stimulation stained for phalloidin (actin filaments) and DAPI (nucleus); scale bar, 20 µm. **B.** Cell spreading area normalised to macrophages on soft hydrogel without LPS stimulation. **C.** Projected nuclear area normalised to macrophages on soft hydrogel without LPS stimulation. **D.** Quantitation of m^6^A in macrophages on soft or stiff hydrogel with/without LPS stimulation; data are for six independent experiments. **E-I.** RT-qPCR quantitation of pro-inflammatory genes (*Il-1β, Il-6, Nos2, Ptgs2*, and *Tnfα*) of macrophages on soft or stiff hydrogel with/without LPS stimulation; data are for three independent experiments. **J.** Protein expression of pro-inflammatory factors (iNOS, p-p65, and p65) in macrophages on soft or stiff hydrogel with/without LPS stimulation. GAPDH was regarded as the endogenous reference. **K.** Expression of nuclear protein (HDAC3) and presence of H3K36me2 in macrophages on soft or stiff hydrogel with/without LPS stimulation. H3 is regarded as the endogenous nuclear reference. **L-M.** Colour-coded representative images of LPS-treated macrophages on soft or stiff hydrogel stained for H3K36me2 (L), HDAC3 (M), and DAPI; scale bar, 20 µm. **N.** Scheme showing the effect on the macrophage structure and the expression of HDAC3 and level of H3K36me2. *p < 0.05, **p < 0.01; ***p < 0.001; ****p < 0.0001 by one-way ANOVA with posthoc multiple comparisons (Tukey's HSD test).

To analyse the effects of stiffness on m^6^A modification and macrophage inflammatory activation, we first constructed stiff and soft gelatine methacryloyl (GelMA) hydrogel (stiff hydrogel: 20% GelMA; soft hydrogel: 8% GelMA) (**Figure S1**). We then analysed the cell morphology of macrophages and found that unlike unstimulated cells, activated macrophages had a flattened shape with a distinctive 'fried-egg' morphology. Moreover, the stiff hydrogel expanded the cell spreading area of the macrophages more than did the soft hydrogel (**Figure 1A-B**). As cell and nuclear morphologies are closely coupled in macrophages 11, a similar expansion trend was observed in the cellular nuclear projection area, suggesting that the hydrogel stiffness has a positive effect on the nuclear projection area (**Figure 1C**). In addition, the stiff hydrogel exhibited higher global levels of m^6^A modification at baseline and under lipopolysaccharide (LPS) stimulation (**Figure 1D**). We subsequently confirmed that stiffness enhanced the inflammatory expression by examining pro-inflammatory markers. *Il-1β*,* Il-6*,* Nos2*,* Ptgs2* and *Tnf-α* were highly expressed under stimulation by the stiffer hydrogel (**Figure 1E-I**). Additionally, inflammatory pathway analysis showed that the nuclear factor (NF)κB pathway was activated via the phosphorylation of p65, demonstrating that the stiffer hydrogel activated the inflammatory pathway (**Figure 1J**).

H3K36me2 is a marker of chromatin compaction and inhibits gene expression 21. In contrast, the presence of HDAC3 is recognised as a marker of loosely packed chromatin that also affects inflammatory activation in macrophages 22, 23. Therefore, we examined the general expressions of HDAC3 and H3K36me2, determining that stiffer hydrogel slightly enhanced the expression of HDAC3 and decreased the expression of H3K36me2 (**Figure 1K-M**). The results show that the stiffness-controlled macrophage inflammatory response is modulated by the effect of nuclear compaction and can be regulated by modifying levels of H3K36me2 and HDAC3 (**Figure 1N**).

### m^6^A-modified *Socs1* as the hub in the stiffness-controlled macrophage inflammatory response

**Figure 2:**
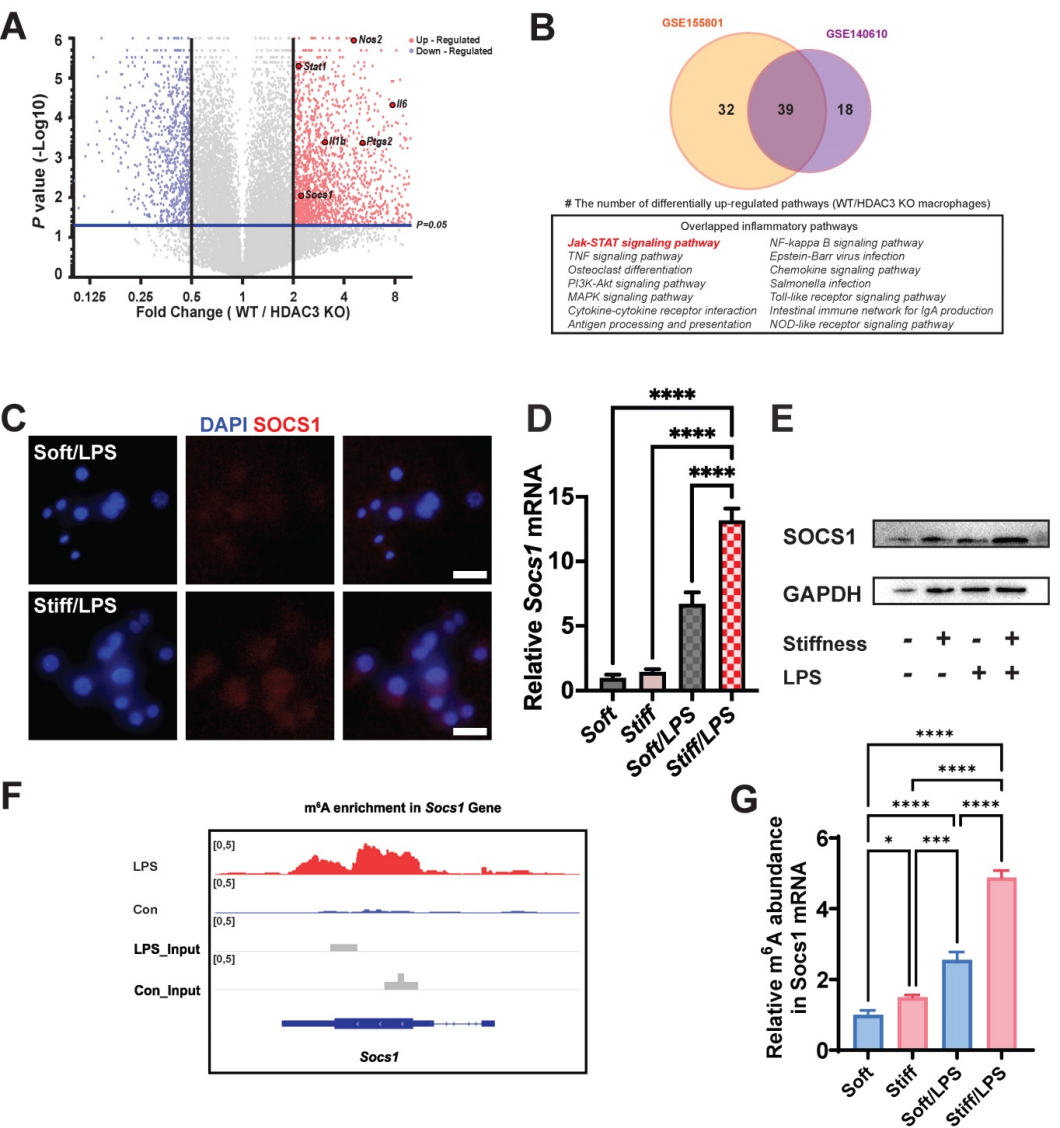
** Stiffness targets SOCS1 to control the macrophage inflammatory response. A.** Volcano plot showing that *Nos2, Il-1β, Il-6, Stat1*, and *Socs1* are among the most up-regulated pro-inflammatory genes in WT macrophages versus HDAC3-KO macrophages (GSE140610). **B.** Venn plot illustrating the overlapping significantly upregulated KEGG pathways in WT macrophages versus HDAC3-KO macrophages between the GSE155801 dataset and the GSE140610 dataset. **C.** Colour-coded representative images of LPS-treated macrophages on soft or stiff hydrogel stained for SOCS1 and DAPI; scale bar, 20 µm. **D.** RT-qPCR quantitation of *Socs1* on soft or stiff hydrogel with/without LPS stimulation; data are for three independent experiments. **E.** Protein expression of SOCS1 in macrophages on soft or stiff hydrogel with/without LPS stimulation. GAPDH was regarded as the endogenous reference. **F.** m^6^A signal density in Socs1 transcript in macrophages with/without LPS stimulation. **G.** MeRIP-qPCR assay showing the effect of stiffness on the m^6^A levels in *Socs1* mRNA in macrophages; data are for three independent experiments. *p < 0.05, **p < 0.01; ***p < 0.001; ****p < 0.0001 by one-way ANOVA with post-hoc multiple comparisons (Tukey's HSD test).

As the stiffness-controlled macrophage inflamematory response is regulated via HDAC3-associated loosening of chromatin, we considered that analysis HDAC3 might help us elucidate the underlying mechanism of the response. We performed data mining on existing Gene Expression Omnibus (GEO) datasets (**GSE140610**) to determine the function of HDAC3 on related genes by using HDAC3 KO macrophages 24. As HDAC3 can directly modulate the inflammatory expression, it is not surprising that most inflammation genes were downregulated in the HDAC3 KO group compared with the wild-type (WT) group. We discovered that *Socs1*, an inflammatory regulatory factor in negative inflammatory feedback, was also HDAC3-dependent (**Figure 2A**). We then investigated the associated pathways via Kyoto Encyclopedia of Genes and Genomes (KEGG) analysis. To precisely target the altered pathways, we included another dataset (**GSE155801**) for the investigation of the macrophage inflammatory response in HDAC3 KO macrophages 25. We discovered that several inflammatory pathways overlapped in the datasets; these included a Socs1-related pathway, namely the Janus kinase (Jak)-signal transducer and activator of transcription (STAT) pathway (**Figure 2B**). We then performed a hierarchical cluster analysis of Gene Ontology (GO) terms via Metascape. The identified top GO terms were determined to have roles in inflammatory association and negative inflammatory modulation (**Figure S2**). The other biological relationship among the selected GO terms revealed a close interrelation of inflammatory clusters (**indicated by the red frame in Figures S3-4**). As stiffness directly affects the levels of HDAC3, we hypothesised that stiffness-controlled *SOCS1* regulation involve the Jak-STAT pathway.

To test this hypothesis, we assessed the *Socs1* expression under various stiffnesses. Higher stiffness directly enhanced the expression of *Socs1* mRNA and SOCS1 protein (**Figure 2C-E**). As the level of m^6^A was globally higher under the higher stiffness condition, we investigated the levels of m^6^A in *Socs1* mRNA. Another dataset (**GSE162254**) for investigating the role of LPS in m^6^A abundance in macrophages was analysed, which revealed there was a higher m^6^A in LPS-stimulated macrophages (**Figure 2F**) 26. We next examined the effect of stiffness on m^6^A methylation in *Socs1* mRNA via methylated RNA immunoprecipitation (MeRIP)-qPCR. The data show that *Socs1* m^6^A methylation was enhanced in the stiffer microenvironment at baseline and in LPS-treated macrophages (**Figure 2G**).

### SOCS1 sustains stiffness-controlled inflammation via a negative feedback mechanism

**Figure 3:**
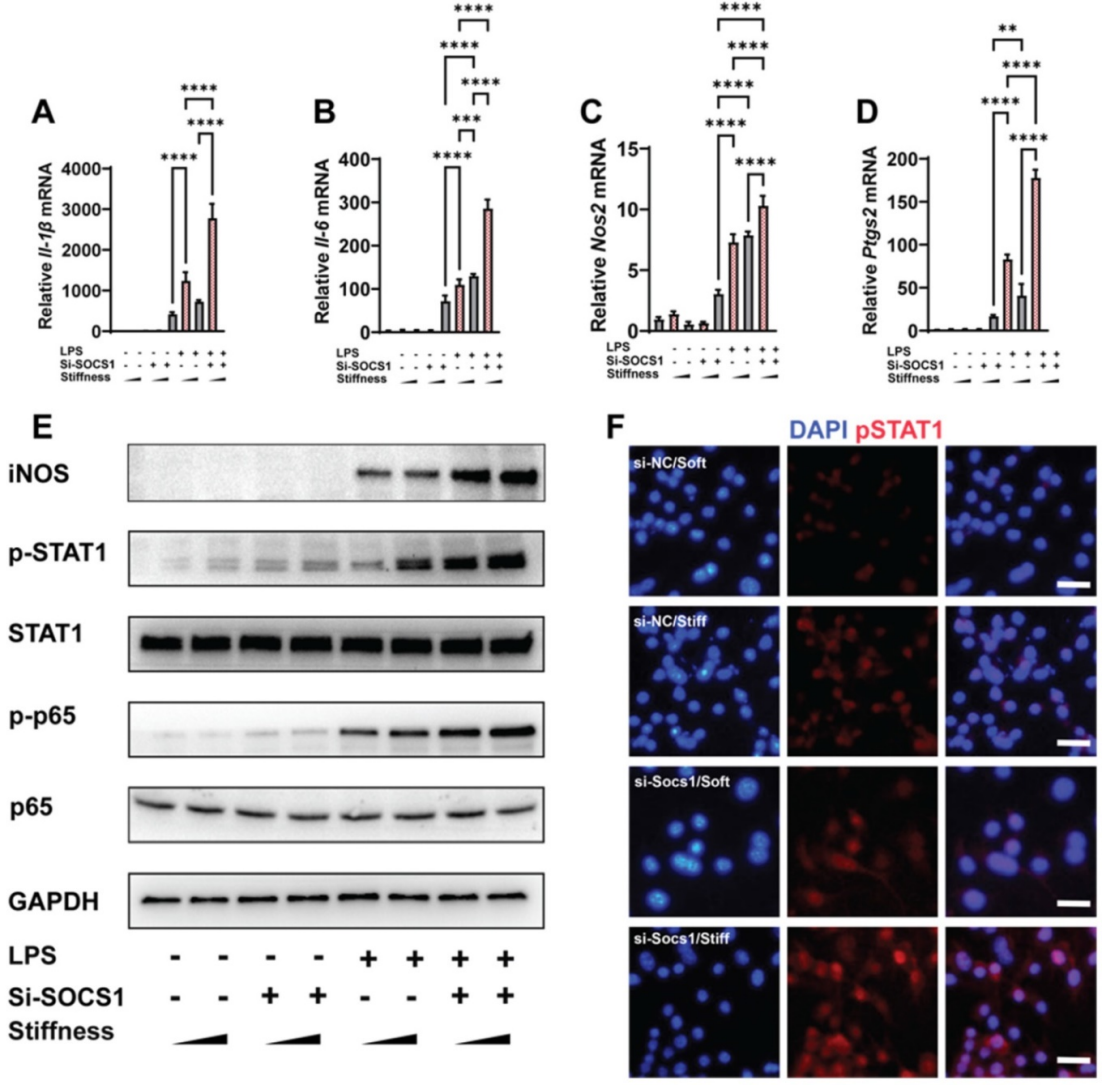
** SOCS1 negatively controls the stiffness-associated macrophage inflammatory response. A-D.** RT-qPCR quantitation illustrating the effect of siRNA Socs1 on the expression of pro-inflammatory genes of macrophages on soft or stiff hydrogel with/without LPS stimulation, namely *Il-1β* (A), *Il-6* (B), *Nos2* (C), and *Ptgs2* (D); data are for three independent experiments. **E.** Western blot showing the effect of si-SOCS1 on the protein expression of pro-inflammatory factors and pathways (iNOS, pSTAT1, STAT1, p-p65, and p65) in macrophages on soft or stiff hydrogel with/without LPS stimulation. GAPDH was regarded as the endogenous reference. **F.** Colour-coded representative images of LPS-treated macrophages on soft or stiff hydrogel stained for pSTAT1 and DAPI; scale bar, 20 µm. *p < 0.05, **p < 0.01; ***p < 0.001; ****p < 0.0001 by one-way ANOVA with posthoc multiple comparisons (Tukey's HSD test).

As SOCS1 was stiffness dependent, we examined how SOCS1 functioned under stiffness stimulation. We knocked down SOCS1 and then examined how this affected inflammatory activation; this revealed that the knockdown of SOCS1 further enhanced the stiffness-induced activity of pro-inflammatory cytokines. Specifically, we observed there was higher expression of *Il-1β*, *Il-6*, *Nos2* and *Ptgs2* in cells treated with the small interfering RNA (siRNA) si-SOCS1 compared with the cells treated with si-scramble (**Figure 3A-D**). Analysis of the inflammatory pathway of NFκB showed it was activated via the phosphorylation of p65 under SOCS1 inhibition. Additionally, the expression of Inducible nitric oxide synthase (iNOS), a marker for the macrophage inflammatory response, was upregulated (**Figure 3E**). As SOCS1 is the regulator of STAT1 and was determined to participate in the Jak-STAT pathway, it is unsurprising that the knockdown of SOCS1 triggered the phosphorylation of STAT1 (**Figure 3F**). In summary, the results show that SOCS1 negatively modulates the macrophage inflammatory response and stiffness sensing via a negative feedback loop involving the Jak-STAT and NFκB pathways.

### FTO is the target demethylase in the stiffness-controlled macrophage inflammatory response

**Figure 4:**
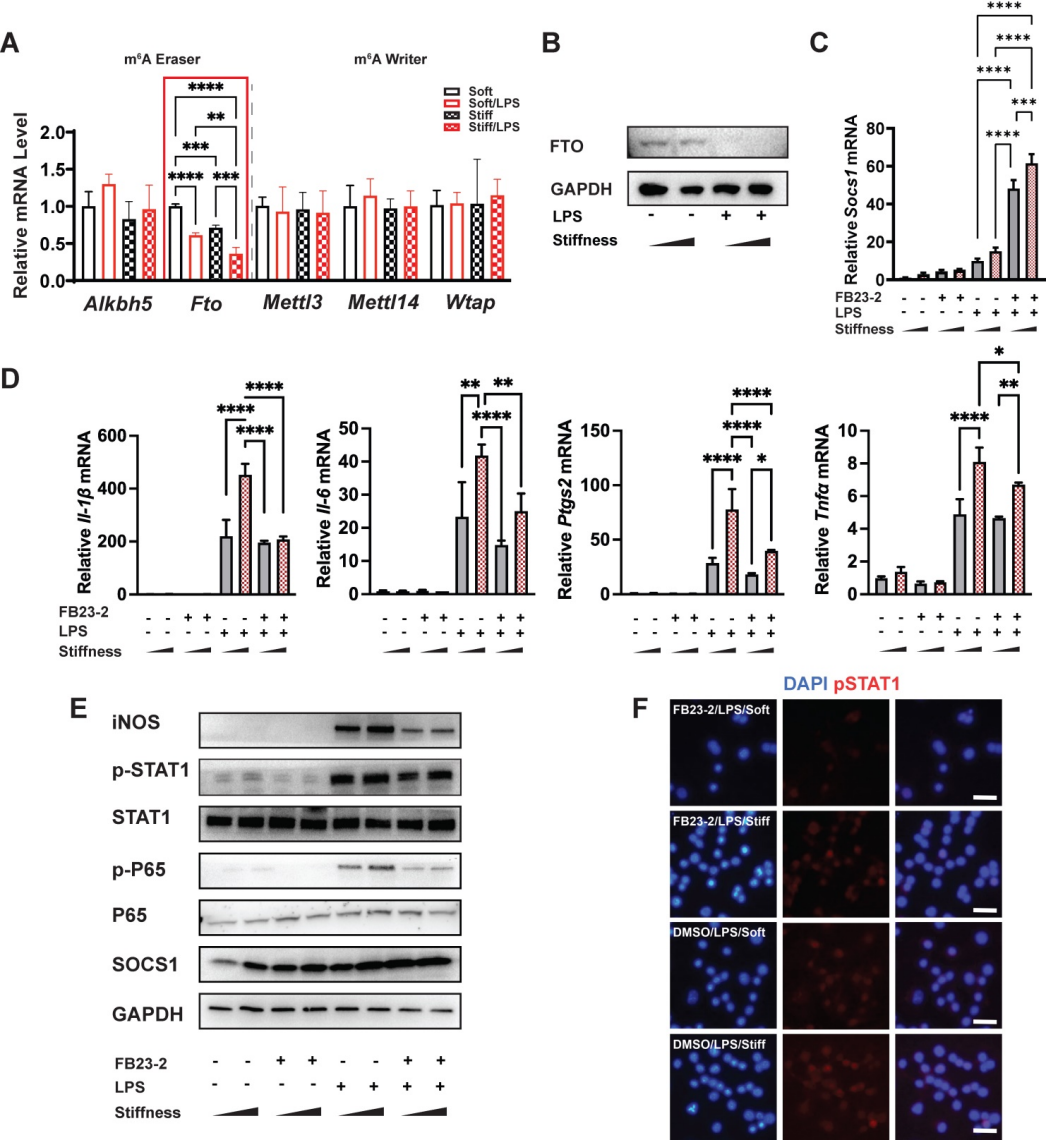
** FTO is required for the stiffness-associated macrophage inflammatory response. A.** RT-qPCR quantitation screening the effect of stiffness on m^6^A-associated enzymes: methyltransferases (m^6^A writers: *Mettl3, Mettl14*, and *Wtap*) and demethylases (m^6^A erasers: *Alkbh5* and *Fto*). A red frame highlights fto, showing its stiffness dependence; data are for three independent experiments. **B.** Western blot showing the effect of stiffness on FTO expression in macrophages on stiff/soft hydrogel with/without LPS treatment; data are for three independent experiments. **C.** RT-qPCR quantitation illustrating the effect of FTO inhibitor FB23-2 on the expression of *Socs1* transcripts under the influence of stiffness in the RAW267.4 cell line. **D.** RT-qPCR quantitation illustrating the effect of FB23-2 on the expression of pro-inflammatory genes (*Il-1β, Il-6, Nos2, Ptgs2*, and *Tnfα*) of macrophages on soft or stiff hydrogel with/without LPS stimulation in a RAW267.4 cell line. **E.** Western blot showing the effect of FB23-2 on the protein expression of pro-inflammatory factors and pathways (iNOS, pSTAT1, STAT1, p-p65, p65, and SOCS1) in macrophages on soft or stiff hydrogel with/without LPS stimulation. GAPDH was regarded as the endogenous reference. **F.** Colour-coded representative images of LPS-treated macrophages on soft or stiff hydrogel stained for pSTAT1, and DAPI; scale bar, 20 µm. *p < 0.05, **p < 0.01; ***p < 0.001; ****p < 0.0001 by one-way ANOVA with post-hoc multiple comparisons (Tukey's HSD test).

Our data demonstrated there are higher levels of m^6^A in stiffer microenvironments than less stiff environments during the macrophage inflammatory response; however, the underlying molecular mechanism of this biochemical pattern remained unclear. As methyltransferases (writers) and demethylases (erasers) affect the levels of m^6^A, we quantified the expression of the m^6^A-critical methyltransferases (*Mettl3*,* Mettl14*, and *Wtap*) and demethylases (*Alkbh5* and* Fto*)*.* The expression of *Fto* reflected the stiffness-controlled macrophage inflammatory response (**Figure 4A**). Similarly, higher stiffness acted synergistically with LPS stimulation to further reduce levels of FTO protein (**Figure 4B**). This might partially explain why the stiff hydrogel systemically and locally (i.e., in *Socs1* mRNA) upregulated levels of m^6^A.

To verify the function of FTO, we first blocked it with its inhibitor (FB23-2). Interestingly, the inhibition of FTO promoted the expression of *Socs1* but suppressed inflammation-related expression (including that of *Il-1β*, *Il-6*, *Nos2*, and *Ptgs2* (**Figure 4C-D**)) and the Jak-STAT and NFκB pathways by decreasing the phosphorylation of STAT1 and p65 (**Figure 4E**). Additionally, the immunofluorescence image of the effect of FTO on the level of pSTAT1 confirmed that stiffness enhanced the phosphorylation of STAT1, but that the inhibition of FTO blocked phosphorylation (**Figure 4F**). Furthermore, the inflammatory response was blocked after the inhibition of FTO in bone-marrow-derived macrophages (BMDMs), as FB23-2 directly suppressed the expression of *Il-1β* and *Il6* (**Figure S5**).

### FTO enhances cell spreading and nuclear projection in microenvironments of various stiffness

**Figure 5:**
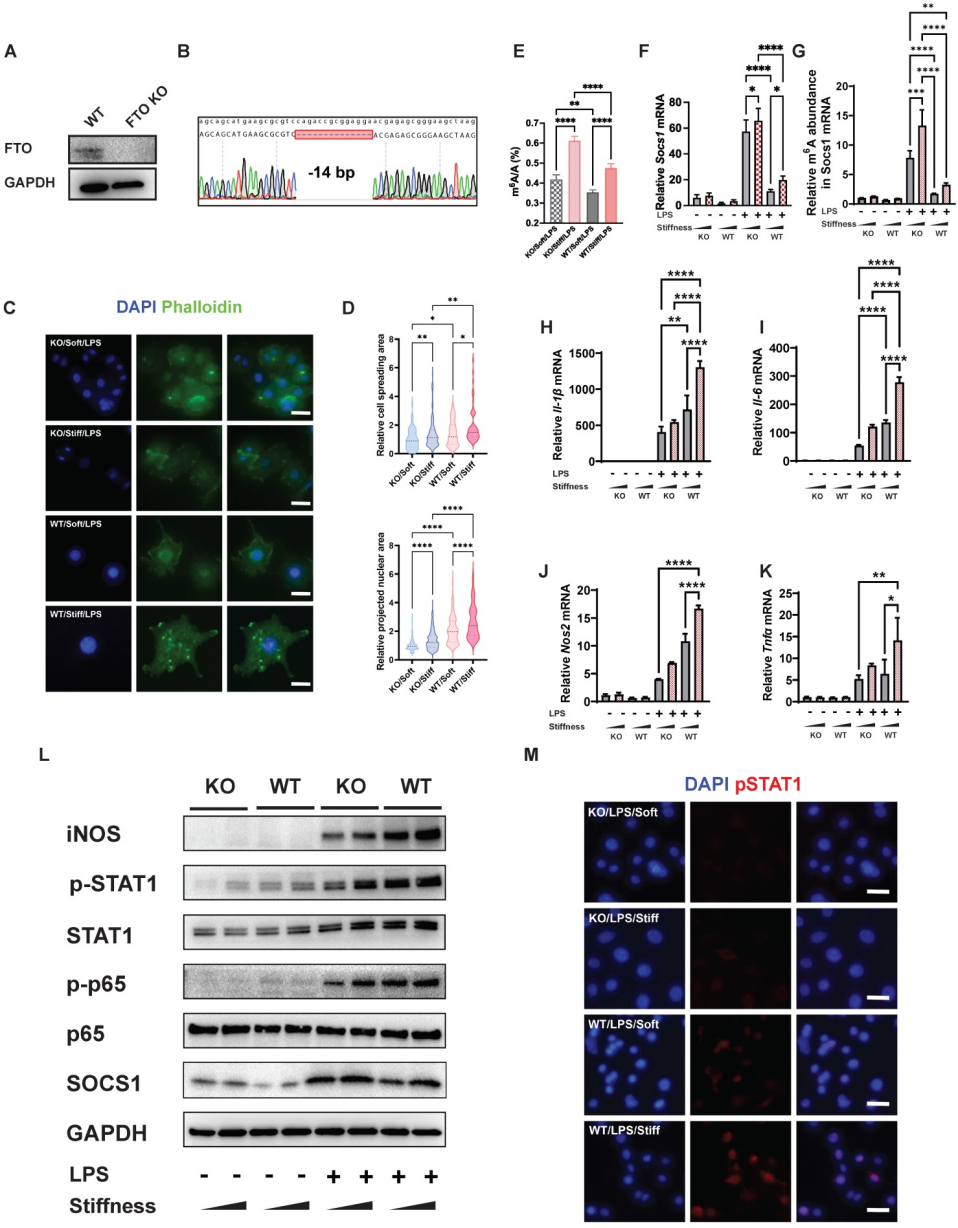
** FTO further enhances the stiffness-associated macrophage inflammatory response via regulating cell spreading and chromatin compaction and increasing the levels of m^6^A in *Socs1* mRNA. A.** Western blot verifying the successful CRISPR KO of the Fto gene in RAW264.7 macrophages. **B.** Sanger sequencing further verifying the successful CRISPR KO of the Fto gene in RAW264.7 macrophages. The deletion of 14 base pairs is observed. **C.** Representative images of LPS-treated macrophages on soft or stiff hydrogel stained with phalloidin (actin filaments) and DAPI (nucleus); scale bar, 20 µm. **D.** Above: cell spreading area normalised to FTO KO macrophages on soft hydrogel; below: projected nuclear area normalised to FTO KO macrophages on soft hydrogel. **E.** Quantitation of m^6^A in LPS-treated macrophages on soft or stiff hydrogel; data are for six independent experiments. **F.** RT-qPCR quantitation illustrating the effect of FTO-KO on the expression of Socs1 transcripts under the effect of stiffness; data are for three independent experiments. **G.** MeRIP-qPCR assay illustrating the effect of FTO-KO on the level of m^6^A of *Socs1* mRNA in macrophages; data are for three independent experiments. **H-K.** RT-qPCR quantitation illustrating the effect of FTO-KO on the expression of pro-inflammatory genes of macrophages on soft or stiff hydrogel with/without LPS stimulation, namely *Il-1β* (H), *Il-6* (I), *Nos2* (J), and *Tnfα* (K); data are for three independent experiments. **L.** Western blot showing the effect of FTO-KO on the protein expression of pro-inflammatory factors and pathways (iNOS, pSTAT1, STAT1, p-p65, p65, and SOCS1) in macrophages on soft or stiff hydrogel with/without LPS stimulation. GAPDH was regarded as the endogenous reference. **M.** Colour-coded representative images of LPS-treated WT and FTO-KO macrophages on soft or stiff hydrogel stained for pSTAT1 and DAPI; scale bar, 20 µm. *p < 0.05, **p < 0.01; ***p < 0.001; ****p < 0.0001 by one-way ANOVA with posthoc multiple comparisons (Tukey's HSD test).

As described above, stiffness enhances cell spreading and nuclear projection and regulates the macrophage inflammatory response via the inhibition of FTO. We thus considered whether the function of FTO directly affects cellular morphology. We constructed an FTO KO macrophage cell line (RAW264.7 cell line) using the CRISPR/Cas9 system. After single-cell selection, western blotting, and Sanger sequencing verification, we obtained an FTO KO cell line with a 14-base-pair deletion in Fto exon 1 (**Figure 5A-B**). Subsequently, we used LPS to activate the FTO KO macrophages. We then investigated the effect of stiffness through quantification of the inflammatory factors and staining the cytoskeleton and chromatin using phalloidin and 4′,6-diamidino-2-phenylindole (DAPI), respectively (**Figure 5C**). Compared with normal cells, the FTO KO cells had smaller cell spreading areas and nuclear projection areas in microenvironments and showed a stiffness-dependence (**Figure 5D**).

### FTO reduces systemic and local levels of m^6^A and enhances the stiffness-controlled macrophage inflammatory response

We next tested the effect of FTO on systemic and local levels of m^6^A. A quantitative analysis of systemic levels of m^6^A showed that the macrophage inflammatory response associated with high levels of m^6^A was enhanced by higher stiffness and significantly inhibited by FTO KO. This effect was also regulated by stiffness; in FTO KO macrophages and WT macrophages, stiff hydrogel always caused higher global levels of m^6^A than soft hydrogel (**Figure 5E**). As Socs1 exerts a negative feedback effect on inflammation, we analysed the effect of FTO knockout on *Socs1*. Socs1 genes were stimulated in FTO KO macrophages, and the stiff microenvironment synergistically facilitated the expression of *Socs1* (**Figure 5F**).

We then investigated the levels of m^6^A in *Socs1* mRNA. Like the global levels of m^6^A, the levels of m^6^A in *Socs1* mRNA were stiffness controlled, as they increased in the stiff microenvironment and decreased in response to FTO KO. At baseline, FTO KO directly (albeit moderately) increased the levels of m^6^A. FTO KO macrophages also had a significantly greater response to LPS stimulation than WT macrophages **(Figure 5G)**.

In contrast, expression of the pro-inflammatory genes (*Il-1β*,* Il-6*,* Nos2*, and *Ptgs2*) was suppressed in the FTO KO macrophages, which was the opposite of the expression trend for *Socs1* (**Figure 5H-K**). *Socs1* participated in the Jak-STAT and NFκB pathways, and we thus investigated the phosphorylation levels of STAT1 and p65. Correspondingly, FTO KO macrophages exhibited increased *Socs1* expression but decreased STAT1 and p65 phosphorylation, and this effect was also regulated by stiffness (**Figure 5L**). The immunofluorescence images verified that FTO acted synergistically with the stiff hydrogel to increase the phosphorylation of STAT1 (**Figure 5M**). In conclusion, the FTO inhibitor and FTO KO experiments both demonstrated that FTO is controlled by stiffness and globally and locally reduces levels of m^6^A to modulate the macrophage inflammatory response.

### Stiffness limits H3K36me2 modification and enhances HDAC3 modulation via the alteration of Fto expression

**Figure 6:**
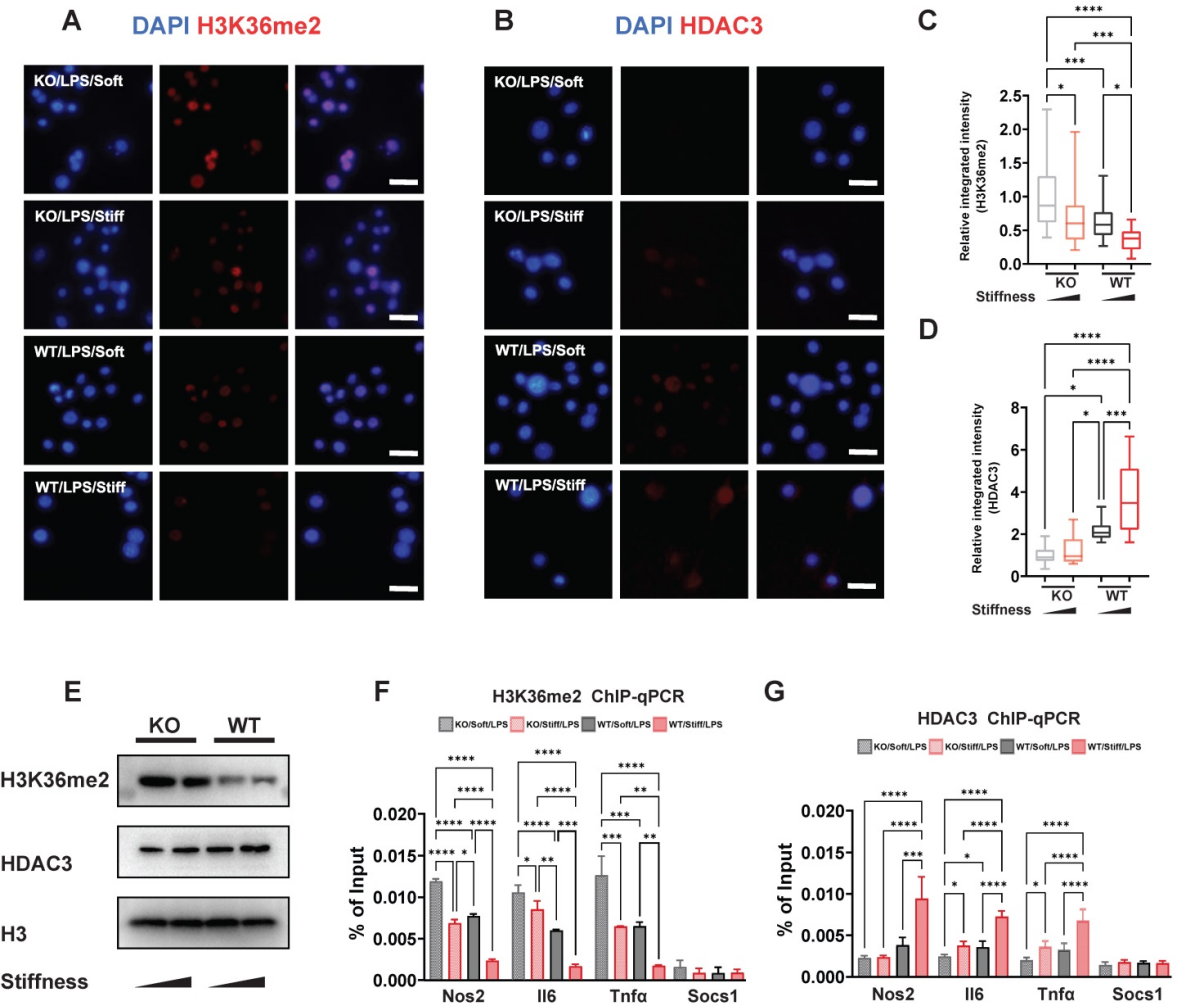
** FTO-KO regulates epigenetic modification via stiffness sensing. A-B.** Colour-coded representative images of LPS-treated wild-type (WT) and FTO-KO macrophages on soft or stiff hydrogel stained for H3K36me2 (A), HDAC3 (B), and DAPI; scale bar, 20 µm. **C-D.** Box plots of the levels of immunofluorescence intensity of H3K36me2 (C) and HDAC3 (D), normalised to FTO-KO macrophages on soft hydrogel. **E.** Western blot showing the effect of FTO-KO on the protein expression of pro-inflammatory factors and pathways (iNOS, pSTAT1, STAT1, p-p65, p65, and SOCS1) in LPS-treated macrophages on soft or stiff hydrogel. **F.** ChIP-qPCR analysis of the effect of FTO-KO on H3K36me2 in the promoter region of Nos2, Il6, Tnfα, and Socs1 genes in LPS-treated macrophages. **G.** ChIP-qPCR analysis of the effect of FTO-KO on the presence of HDAC3 in the promoter region of Nos2, Il6, Tnfα, and Socs1 genes in LPS-treated macrophages; data are for three independent experiments. *p < 0.05, **p < 0.01; ***p < 0.001; ****p < 0.0001 by one-way ANOVA with posthoc multiple comparisons (Tukey's HSD test).

Chromatin structure and inflammatory expression have been attributed to histone modification via markers such as H3K36me2 and enzymes such HDAC3. We showed that stiffness is negatively correlated with levels of H3K36me2 but positively correlated with levels of HDAC3, and that FTO directly enhances inflammatory activation and changes the cellular morphology. We therefore supposed that FTO also participates in the process of histone alteration. To confirm this, WT and FTO KO macrophages exposed to various stiffness were stained for H3K36me2 and HDAC3. As we showed previously, stiffness oppositely affects H3K36me2 and HDAC3; however, FTO KO further limits the levels of HDAC3 and induces H3K36me2 (**Figure 6A-D**). These results were verified through western blotting; the total levels of H3K36me2 and HDAC3 exhibited the same trend as the immunofluorescence staining results (**Figure 6E**). Furthermore, chromatin immunoprecipitation (ChIP)-qPCR experiments revealed there were higher levels of HDAC3 in the Il-6, Nos2, and Tnf-α gene promoter regions, which are regulated by stiffness and FTO. The enrichment of H3K36me2 had the opposite trend (**Figure 6F-G**). However, no significant modifications were detected at the Socs1 gene promoter (**Figure 6F-G**), suggesting that other epigenetic modifications and mechanisms are involved in Socs1 regulation.

### Stiffness controls the macrophage inflammatory response via the m^6^A reader YTHDF1

**Figure 7:**
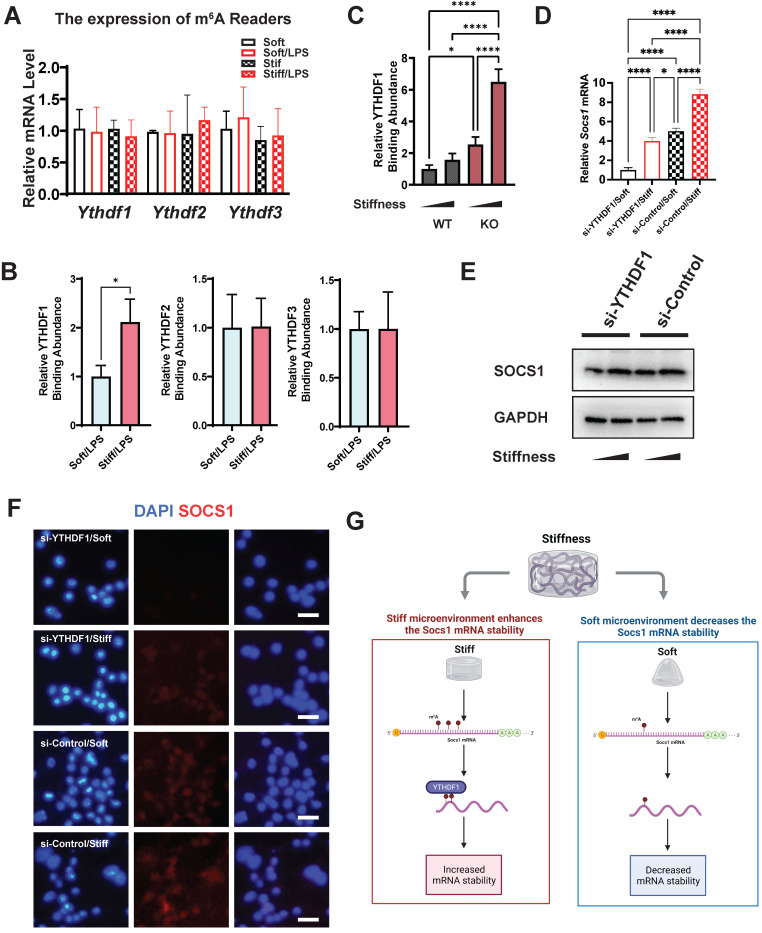
** YTHDF1 is the m^6^A reader, which regulates SOCS1 expression via stiffness sensing. A.** RT-qPCR quantitation screening the effect of stiffness on the gene expression of m^6^A readers (*Ythdf1, Ythdf2*, and *Ythdf3*); data are for three independent experiments. **B.** RIP-qPCR quantitation screening the effect of stiffness on the quantity of m^6^A readers (YTHDF1, YTHDF2, and YTHDF3) and *Socs1* mRNA complex; data are for three independent experiments. **C.** RIP-qPCR quantitation illustrating the effect of FTO-KO on the quantity of YTHDF1 and *Socs1* mRNA complex; data are for three independent experiments. **D.** RT-qPCR quantitation illustrating the effect of si-YTHDF1 on the expression of *Socs1* transcripts under the effect of stiffness; data are for three independent experiments. **E.** Western blot showing the effect of si-YTHDF1 on SOCS1 expression in macrophages on stiff/soft hydrogel. **F.** Colour-coded representative images of LPS-treated WT and FTO-KO macrophages on soft or stiff hydrogel stained for SOCS1 and DAPI; scale bar, 20 µm. **G.** Scheme showing the effect of stiffness on the YTHDF1 and Socs1 mRNA complex, created with BioRender.com. For two-group comparison, a Student's *t*-test was applied for testing the significance with *p < 0.05; for comparison with groups (>2), a one-way ANOVA with post-hoc multiple comparisons (Tukey's HSD test) was used for testing significance. *p < 0.05, **p < 0.01; ***p < 0.001; ****p < 0.0001.

The m^6^A readers are another indispensable element in understanding the regulation of m^6^A levels. We therefore analysed the gene expression of the m^6^A readers (*Ythdf1*,* Ythdf2*, and *Ythdf3*), which revealed no significant alteration in their expression (**Figure 7A**). We then investigated the abundance of the readers in *Socs1* mRNA via RNA immunoprecipitation (RIP). The RIP-qPCR results show that only YTHDF1 was abundant at the sites with abundant m^6^A binding (**Figure 7B**). We then analysed the correlation between FTO and YTHDF1; this showed that the binding of YTHDF1 in *Socs1* mRNA was stiffness dependent, and FTO KO further enhanced the abundance of YTHDF1 in *Socs1* mRNA (**Figure 7C**). These discoveries suggest that YTHDF1 is the key reader involved in the stiffness-dependent macrophage inflammatory response that operates via Fto. As YTHDF1 recognises the m^6^A sites in *Socs1* mRNA, we speculated that the downregulation of YTHDF1 might affect the function of *Socs1* in stiff microenvironments. Owing to that YTHDF1 functions to increase mRNA stability, we first assessed the decay of Socs1 mRNA. We found that a stiff microenvironment extended the half-life of *Socs1* mRNA (**Figure S6**), and the extension trend was similar to that of the abundance of YTHDF1 in *Socs1* mRNA. To further analyse the role of YTHDF1 in the stiffness-dependent macrophage inflammatory response, we inhibited YTHDF1 expression in macrophages via treatment with an siRNA (si-YTHDF1), which revealed that the regulation of *Socs1* in stiff microenvironments was YTHDF1 dependent. Compared with the WT cells, the YTHDF1-downregulated cells had decreased levels of *Socs1* mRNA and SOCS1 protein (**Figure 7D-F**). These observations suggest that the reader YTHDF1 functions as a critical regulator during the stiffness-dependent macrophage inflammatory response.

## Discussion

Microenvironments activate macrophages in different ways, with one of the most important microenvironment-mediated activation processes involving the alteration of cell morphology 3. Macrophage elongation is correlated with M2 polarisation, which may facilitate tissue healing 9, and macrophage spreading is associated with M1 polarisation, which accelerates the inflammatory response 8, 9, 11. Moreover, macrophage deformation affects the degree of nuclear chromatin condensation; elongation increases chromatin condensation, whereas enlarging the spreading area reduces chromatin condensation and increases nuclear projection 11, 27. These nuclear alterations are modulated by histone modifications; levels of H3K36me2 are positively correlated with condensed chromatin and levels of HDAC3 are positively correlated with the loosening chromatin 21-23. In the present study, we investigated the stiffness of macrophages and how stiffness mediates the morphological alterations in cell shape and chromatin condensation. We discovered that high stiffness enhanced the macrophage inflammatory response, levels of m^6^A and directly increased the cell spreading area of macrophages with loosened chromatin. This alteration of cell morphology was related to HDAC3 and H3K36me2, which are a chromatin-modifying enzyme and chromatin modification, respectively. Stiffness positively regulated the cell spreading area and chromatin loosening, during which the expressions of pro-inflammatory genes was dependent on the presence of H3K36me2 and the binding of HDAC3. Additionally, our results indicate there was a small but clear alteration of the expression of HDAC3 and occurrence of H3K36me2, which we inferred as meaning that supposed that a cascade of epigenetic modifications modulate macrophage activation. SOCS1 was also found to participate in negative-feedback regulation of the stiffness-dependent macrophage inflammatory response.

Unlike pro-inflammatory gene expression, SOCS1 expression did not show dependence on the levels of HDAC3 and H3K36me2, which suggests its expression is modulated by different epigenetic mechanisms. The present study did not focus on the correlation between levels of HDAC3 and H3K36me2 and their effects on Socs1, and thus we cannot rule out that HDAC3 and H3K36me2 play a role in this regard. These open questions are interesting and deserve future research. Further analysis of the levels of m^6^A in *Socs1* mRNA revealed that the level of m^6^A in* Socs1* exhibited a similar trend to the global levels of m^6^A. We also observed that the levels of m6A were higher in a stiff microenvironment at baseline or under LPS stimulation. As the regulation of m^6^A depends on methyltransferases and demethylase, we screened the expression of *Fto*,* Wtap*,* Mettl3*,* Mettl14*, and *Alkbh5* and found that Fto was the only gene whose expression depended on both stiffness and inflammation. Subsequently, we generated FTO KO macrophages to validate the function of FTO. Interestingly, FTO KO macrophages had a lower cell-spreading area with condensed chromatin than WT macrophages. FTO also participated in the formation of m^6^A in *Socs1*; at baseline and under LPS stimulation, the FTO KO macrophages had a higher level of m6A than the WT macrophages. In addition, FTO modulated the negative feedback process, which highlights the essential role of FTO in this process.

The mammalian body is complex, due to its various tissue environments 3. Macrophages play a vital role in nonspecific immunity, as they defend the body against outside invasion 28. Macrophages are diffusely scattered throughout the body and are named depending on whether they are located in, for example, the liver (Kupffer cells), spleen and lymph nodes (sinus histiocytes), lungs (alveolar macrophages), or central nervous system (microglia). Stiffness varies between tissues, ranging from 0.1 kPa (brain) to over 100 kPa (bone), and thus the functions of macrophages are diverse because of the different microenvironments they inhabit 29. Interestingly, stiffness also plays a crucial role in macrophage functions: Wang et al. showed that compared with soft GelMA (5%), stiff GelMA (15%) promotes M1 polarisation and suppresses M2 polarisation 30. Similarly, Liu et al. found that compared with a soft matrix, exceptionally stiff materials such as glass stimulate the macrophage inflammatory response and enlarge cell-spreading areas 12, 13. Consistent with those studies, we found that stiffness positively alters macrophage cell spreading and chromatin condensation and activates the inflammatory response.

The process of inflammation regulation via stiffness sensing involves complex interactions between various molecules and remains unclear. The most commonly accepted theories regarding mechanical inflammatory activation involve mechanical receptors, such as piezo-type mechanosensitive ion channel component 1 (PIEZO1) and yes-associated protein 1 (YAP). PIEZO1 is an ion channel that regulates its downstream pathway by modulating calcium-ion concentrations inside and outside the cells 31. YAP might participate in nuclear expression via the YAP/TAZ complex 32. Epigenetic modulation is another essential component of mechanical loading regulation, as the acetylation of histone 3 (H3) can integrate with the activity of the proto-oncogene tyrosine-protein kinase Src to alter the inflammatory response 10. In the current study, we extended our understanding of the mechanisms of mRNA modification, demonstrating that the FTO-*Socs1*-YTHDF1 axis is vital to macrophage mechano-sensation and the inflammatory response.

The expression of the FTO gene was discovered to be affected by obesity in 2007 33. Scientists later found that FTO protein catalyses the oxidative demethylation of m^6^A in RNA 34. This modification has been demonstrated to affect disease progression but its underlying mechanisms are unknown. The targeted inhibition of FTO decreases body weight, suppresses cancer development, and inhibit the macrophage inflammatory response 35. Although the RNA-related function of FTO is known to affect gene expression and disease pathology 33, the effects of the microenvironment and cell morphology on the function of FTO were previously unknown. In this study, we demonstrated that the stimulation of macrophages by a stiff hydrogel enlarged the cell spreading area and nuclear projection area, and that this effect was rescued by the deletion of the Fto gene. This indicates that regulatory mechanisms involving stiffness-dependent activation may affect the activation of macrophages via FTO. The fact that FTO mainly affects epigenetic alterations, as does stiffness, highlights the importance of investigating its epigenetic regulatory mechanisms. Given that FTO modulates histone modification of the promoters of inflammatory genes, and that the histone tags are also stiffness associated 8, 18, 20-22, we proposed that an axis links the function of FTO to histone modification and thus leads to inflammatory activation and cellular morphology alteration. We showed that FTO is regulated by stiffness; i.e., higher stiffness suppresses Fto expression. In addition, we found that a stiff microenvironment directly enlarges cells and enhances inflammation, as has been seen in previous studies 30. This suggests that stiffness affects Fto expression via inflammatory activation. Furthermore, as the deletion of FTO had a negative-feedback effect on cell morphology and macrophage inflammatory response, this suggests it is involved in a negatively modulatory loop.

SOCS1 regulates the type-I interferon-induced pathways and participates in LPS-induced Jak-STAT and NFκB pathways via ubiquitin‐mediated proteasomal degradation, thereby acting as the key negative regulator controlling macrophage-mediated inflammation 36. The interaction between SOCS1, p65, and STAT1 inhibits further inflammation by ubiquitinating and degrading TNF receptor-associated factor 6 37. Furthermore, SOCS1 possesses an SH2 domain that enables it to directly interacting with the JAK protein and thus hinder JAK activity 38. Another mechanism involving SOCS1 is the inhibition of kinase activities, including the Jak-STAT pathway, via the kinase inhibitory region 39. m^6^A is also related to the function of SOCS1; i.e., SOCS1 has been determined to be a critical METTL14 target in the regulation of the macrophage inflammatory response. METTL14 can directly bind to *Socs1* mRNA and increase the level of m^6^A. Additionally, FTO mediates m^6^A demethylation in *Socs1* mRNA, affecting the stability of *Socs1* 20. There is evidence suggesting that tissue stiffness induces the expression of the receptor tyrosine kinase Axl, which takes part in STAT1 signalling via SOCS1 suppression, thereby playing a pivotal role in stiffness-associated inflammation 40. From our observations, higher stiffness increases *Socs1* transcription and the level of m^6^A via the inhibition of FTO and regulates the mechanism involved in the Jak-STAT and NFκB pathways. In addition, we found a positive correlation between Socs1 expression and YTHDF1 recognition. YTHDF1 expression did not statistically change in the stiff microenvironment but it significantly increased in the presence of the binding of Socs1 mRNA. A molecule such as YTHDF1 can promote protein synthesis through interactions with translation, while decreasing mRNA degradation, increasing mRNA stability, and improving translation efficiency 20, 41, 42. In stiff microenvironments, YTHDF1 binds to m^6^A, thereby affecting the level of Socs1 expression. Moreover, YTHDF1 and Socs1 interact with one another and regulate macrophage activation in a stiff environment.

In the mononuclear phagocyte system, the functions of macrophages somewhat vary according to their location. Macrophages have a specific effect on the disorder of cells, thereby affecting the occurrence and development of disease. Disorders and varying degrees of inflammatory changes increases the difficulty of diagnosing and treating disease. The formation of m^6^A is the most ubiquitous modification of RNA and thus can directly affect gene expression and hence the pathogenesis of various diseases. Therefore, elucidating the causes and mechanisms that lead to the formation of m^6^A and to the stiffness-mediated macrophage inflammatory response enhances our understanding of systemic inflammation and may facilitate the development of new therapeutic approaches.

## Materials and Methods

### Reagents and resources

The reagent names, brands, identifiers, catalogue numbers, dilution information for antibodies, and primer sequences can be found in the supplementary documents.

### Cell culture

Mouse BMDMs were a gift from Dr. Chen Ling (Li Ka Shing Faculty of Medicine, HKU). BMDMs were cultured in DMEM supplemented with 1% antibiotic solution (100 U/mL penicillin and 0.1 mg/mL streptomycin), 10% (v/v) foetal bovine serum at 37 °C in 5% carbon dioxide and 25 ng/ml mouse M-CSF Protein (M-CSF, R&D Systems, MN, USA). The mouse macrophage cell line RAW264.7 and FTO knockout macrophages were cultured in Dulbecco's modified Eagle's medium (DMEM) supplemented with 1% antibiotic solution (100 U/mL penicillin and 0.1 mg/mL streptomycin) and 10% (v/v) foetal bovine serum at 37 °C in 5% carbon dioxide. For FTO inhibition, macrophages were cultured with 5 μM FB23-2 for 24 h. For LPS stimulation, macrophages were incubated with 100 ng/mL LPS for 6 h, and cell lysates were then prepared for further analysis.

### GelMA hydrogel preparation

Ten grams of gelatine (type A) was added to 100 mL of phosphate-buffered saline (PBS) at 50 °C to give a final concentration of 10% (wt/vol), and the resulting mixture was stirred to facilitate gelatine dissolution. The resulting solution was treated with 12 mL of methacrylic anhydride and then stirred at 50 °C for 4 h. After this time, unreacted methacrylic anhydride was removed by centrifugation (3,500 rpm for 3 min). The remaining mixture was dialysed at 40 °C against deionised water for 7 d. The final lyophilised GelMA was obtained by lyophilisation of the dialysed solution (which typically took 3 days). GelMA hydrogel was synthesised by first preparing the GelMA [soft: 8% (wt/vol); stiff: 20% (wt/vol)]/IC2959 [0.05% (wt/vol)] mixture in PBS and transferring the mixture to a water bath at 37 °C until the mixture dissolved. Then, the hydrogel was obtained by photo-cross-linking the solution in a 365-nm ultraviolet cross-linker for 15 min. Finally, the hydrogel was washed once with PBS to remove traces of the photoinitiator (IC2959).

### Rheological analysis

Rheological analysis was performed using a Kinexus Lab plus system (Malvern Panalytical, Worcestershire, UK). The parameters of the oscillatory time sweep were a gap between the rotor and plate of 0.5 mm, a strain sweep of 0.1-1,000%, and frequency sweep of 0.1-10 Hz.

### Deletion of the Fto gene using the CRISPR/Cas9 system

The FTO KO RAW264.7 cell line (RAW264.7^ΔFTO^) was generated using the CRISPR/Cas9 system **(Figs. 5-6)**. The CRISPR ribonucleoprotein system was obtained from Genscript (Piscataway, NJ, USA), and the guide (g)RNA was designed using the GenCRISPR gRNA design tool (https://www.genscript.com/tools/gRNA-design-tool). For this study, an FTO single gRNA (CATGAAGCGCGTCCAGACCG) was selected based on its on-target, off-target and overall scores. RAW264.7 cells were co-transduced with the single gRNA and Cas9 protein via Lipofectamine® RNAiMAX reagent (Thermo Fisher Scientific, MA, USA). Clonal cell lines were isolated by serial dilution. Finally, genome editing was verified through western blotting and sequencing.

### siRNA-mediated gene knockdown

Validated siRNAs targeting SOCS1 and YTHDF1 (Santa Cruz Biotechnology, Dallas, TX, USA) were applied to silence Socs1 and Ythdf1 gene expression, and a scrambled siRNA (Invitrogen, MA, USA) was used as a negative control. The siRNAs were transfected into RAW264.7 cells using Lipofectamine® RNAiMAX reagent (Thermo Fisher Scientific, MA, USA).

### RT-qPCR

RNeasy Mini kits (Qiagen, Hilden, Germany) were used to extract total RNA. Two thousand nanograms of RNA were used to synthesise the first-strand cDNA via SuperScript™ III Reverse Transcriptase (Thermo Fisher Scientific, MA, USA). RT-PCR was performed using a StepOnePlus™ Real-Time PCR System (96-well plate) or QuantStudio™ 6 Flex Real-Time PCR System (384-well plate, Thermo Fisher Scientific, MA, USA) with TB Green® Premix Ex Taq™ (Tli RNase H Plus; Takara, Shiga, Japan). The target-gene copy numbers were normalised against the copy number of an endogenous housekeeping gene via the 2^-△△Ct^ formula. Final statistics were obtained for three biological replicates, with each biological replicate being the average of three technical replicates. *Gaphd* was used as the endogenous control for normalisation. The PCR primers are listed in Table S1 43, 44.

### Western blotting

Cells were lysed (Thermo Fisher Scientific, MA, USA) via a radioimmunoprecipitation assay buffer (RIPA, Thermo Fisher Scientific, MA, USA) with the Halt™ Protease and Phosphatase Inhibitor Cocktail (Thermo Fisher Scientific, MA, USA). Protein concentrations were determined using a bicinchoninic acid assay kit (Pierce, Rockford, USA) before separation on 4-20% Mini-PROTEAN® TGX™ Precast Protein Gels (Bio-Rad, Hercules, CA, USA) and subsequent transfer onto polyvinylidene difluoride membranes. The blotted membranes were blocked with Pierce™ Fast Blocking Buffer (Thermo Fisher Scientific, MA, USA). Primary [Histone H3 (1:1,000), iNOS (1:1,000), STAT1 (1:1,000), pSTAT (1:1,000), p65 (1:1,000), p-p65 (1:1,000), FTO (1:1,000), SOCS1 (1:1,000), HDAC3 (1:1,000), H3K36me2 (1:1,000), GAPDH (1:1,000)] and secondary antibodies [anti-mouse IgG, HRP-linked antibody (1:3,000), anti-rabbit IgG, HRP-linked antibody (1:3000)] were diluted and used according to the manufacturers' recommendations. WesternBright Quantum or Sirius Chemiluminescent Detection Kits (Advansta, California, USA) were used to visualise the protein bands. Detailed antibody information is listed in Table S2.

### Immunofluorescence

PBS buffer was used to rinse cells once. Then, 4% paraformaldehyde was used to fix the cells for 15 min at room temperature, and the cells were subsequently rinsed three times in PBS for 5 min. Ready-to-use Immunofluorescence Blocking Buffer (Cell Signaling Technology, MA, USA) was applied for 60 min for blocking and permeabilisation. The blocking buffer was aspirated, and the primary antibodies [pSTAT (1:200), SOCS1 (1:200), HDAC3 (1:200), H3K36me2 (1:200)] diluted in ready-to-use Immunofluorescence Antibody Dilution Buffer (Cell Signaling Technology, MA, USA) were applied overnight at 4 °C. Next, the cells were rinsed and incubated in fluorochrome-conjugated secondary antibodies [goat anti-mouse IgG H&L (1:200), goat anti-rabbit IgG H&L (1:200)] for 2 h, and the cytoskeleton was stained with Alexa Fluor® 488 phalloidin, according to the manufacturer's instructions. Finally, Mounting Medium with DAPI (Abcam) was applied to mount samples after rinsing. Immunofluorescence images were captured using a Nikon ECLIPSE Ti-S Fluorescence Inverted Microscope (Nikon, Tokyo, Japan). Detailed antibody information is listed in Table S2.

### m^6^A quantitation

The EpiQuik™ m^6^A RNA Methylation Quantification Kit (Colorimetric; Epigentek, USA) was used to quantify the global levels of m^6^A. Briefly, after obtaining the total RNA, 100 ng RNA samples were added to assay wells and incubated for 90 min at 37 °C. After washing, a capture antibody was added and the resulting samples were incubated for 1 h at room temperature, and then washed and detected with an antibody enhancer solution. Finally, a colour development solution was added, and after 2 to 15 min the absorbances of the samples were measured using a microplate reader at 450 nm.

### MeRIP-qPCR

The EpiQuik™ CUT&RUN m^6^A RNA Enrichment Kit (Epigentek, USA) was used to enrich protein-binding RNAs. First, total RNA was extracted as described above and incubated with an anti-m^6^A antibody in the assay wells. m^6^A-bound mRNA was then fragmented using the Cleavage Enzyme Mix, and the fragments were purified using RNA-binding beads. After washing twice with 90% ethanol, an elution buffer was used to release the RNA from the beads. Finally, qPCR was performed to determine the enrichment of m^6^A-bound mRNA.

### ChIP-qPCR

A ChromaFlash High-Sensitivity ChIP Kit (Epigentek, USA) was used for chromosome immunoprecipitation, following the manufacturer's instructions. In brief, cells (1 × 10^6^ cells per ChIP) were collected from 10-cm dishes and lysed in a lysis buffer. The protein-bound DNA was then sheared and transferred into protein-antibody immunoprecipitation wells [IgG (1:30), HDAC3 (1:30), H3K36me2 (1:30)]. After purifying the protein-DNA complexes and reverse cross-linking, the purified DNA was collected in capture tubes. qPCR was performed to determine the enrichment of protein-bound DNA. Detailed primer and antibody information can be found in Tables S1 and S2 45-47.

### RIP-qPCR

RIP was performed as previously described 48. Briefly, harvested cells (1 × 10^7^ cells per RIP) were obtained and resuspended in a mixture containing distilled water, PBS, and nuclear isolation buffer (4% Triton X-100, 40 mM Tris-HCl pH 7.5, 1.28 M sucrose, 20 mM MgCl_2_). Nuclei were pelleted by centrifugation at 2,500 × *g* for 15 min and then resuspended in 1 mL of RIP buffer [25 mM Tris pH 7.4, 150 mM KCl, 5 mM EDTA, 0.5% NP40, 0.5 mM DTT, 100 U/mL RNAase inhibitor and protease inhibitors]. Processed nuclei were separated into two fractions of 500 μL each (for the mock and actual immunoprecipitation) through mechanical shearing. Centrifugation at 13,000 rpm for 10 min was conducted to pellet the nuclear membrane and debris. Antibodies to YTHDF1 (1:30, Proteintech, IL, USA), YTHDF2 (1:30, Proteintech, IL, USA), and YTHDF3 (1:30, Proteintech, IL, USA) were added to the supernatant and incubated overnight at 4 °C with gentle rotation. Twenty microliters of protein A/G beads were then added to the supernatant, which was subsequently incubated for 1 h at 4 °C with gentle rotation. The beads were pelleted in a magnetic device for 2 min. Afterwards, the supernatant was removed, and the remaining beads were resuspended in 500 μL of RIP buffer. This process was repeated for a total of three RIP washes, followed by one wash in PBS. The beads were resuspended and purified using a TRIzol Plus Purification Kit. qPCR was performed to determine the enrichment of protein-bound RNA. Detailed primer information can be found in Table S1 20.

### mRNA Decay

The macrophages were cultured on hydrogels of varying stiffness. After stimulation with 100 ng/ml LPS for 6 h, the cells were washed once with PBS buffer and then transferred to fresh media containing 10 ug/mL actinomycin D. Following actinomycin D treatment, RNA was extracted, reverse transcribed, and quantified by qPCR. An analysis of changes in mRNA levels was performed at each time point, after normalising to zero hours.

### Data mining

Presented data (**Figure 2A, 2B and 2F**) were mined from GEO datasets: **GSE155801 (RNA-seq)**, **GSE140610 (RNA-seq)**, and **GSE162254 (MeRIP-seq) 24-26, 49**. The Database for Annotation, Visualization and Integrated Discovery (DAVID, https://david.ncifcrf.gov/) v6.8 was used for functional annotation (KEGG) 50, 51. Metascape (https://metascape.org/) was used for functional annotation (GO) and GO network construction 52.

### Statistical analysis

The Integrative Genomics Viewer (IGV, Broad Institute, MA, USA) was used to visualise the peak signalling MeRIP-seq data 53 ImageJ (National Institutes of Health, MD, USA) was used to analyse and quantify the immunofluorescence images 54. A Student's *t*-test with two comparisons and a one-way analysis of variance (ANOVA) with multiple comparisons [Tukey's honestly significant difference (HSD) test] was conducted as a post-hoc test using GraphPad Prism 9 (GraphPad software, CA, USA). Data are presented as means ± standard deviations (SDs). *p* < 0.05 was considered a significant difference.

### Data availability

The sequencing data were obtained from GEO datasets 49. The remaining data in this study are available from the corresponding authors upon reasonable request.

## Supplementary Material

Supplementary figures and tables.
