# Supplementary material for: N6-methyladenosine of Socs1 modulates macrophage inflammatory response in different stiffness environments
Source: Int J Biol Sci. 2022 Sep 11;18(15):5753–69. doi: 10.7150/ijbs.74196 (PMC9576523; doi:10.7150/ijbs.74196)
Supplement: Supplementary file 1 — Supplementary figures and tables. [file ijbsv18p5753s1.pdf]

N6-methyladenosine of Socs1 modulates macrophage inflammatory response in different stiffness environments

Zhekai Hu [1], Yuqing Li [2], Weihao Yuan [3], Lijian Jin [2], Wai Keung Leung [2], Chengfei Zhang [4], Yanqi Yang [1]*

Supplemental Figures and Figure Legends

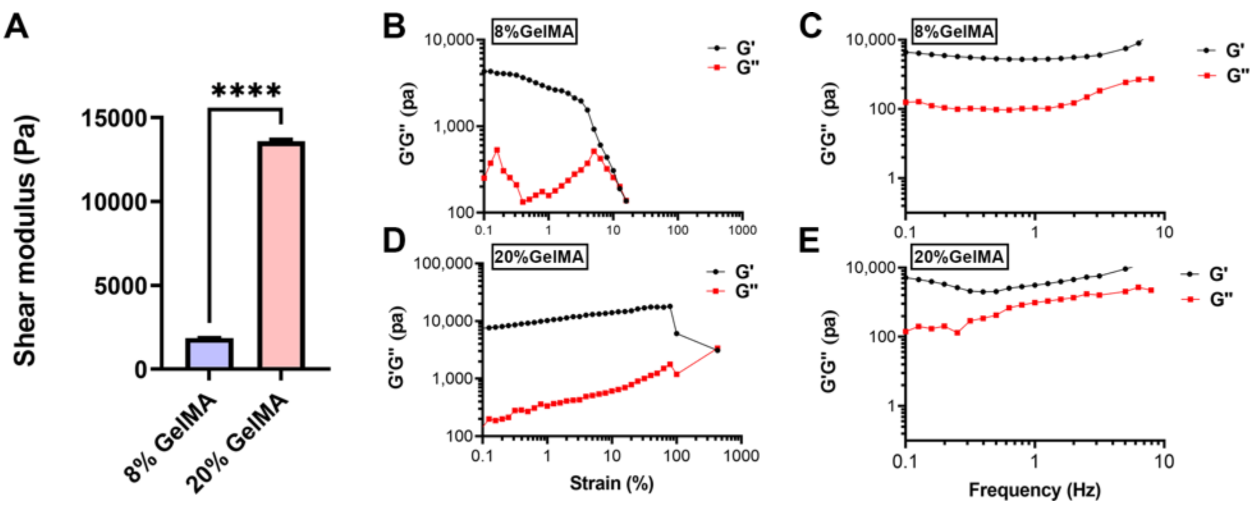

Figure S1. The characteristics of GelMA hydrogel. (A) Stiffness of 8% and 20% GelMA. (B) Correlation between strain and G′ and G′′ of 8% GelMA. (C) Correlation between frequency and G′ and G′′ of 8% GelMA. (D) Correlation between strain and G′ and G′′ of 20% GelMA. (E) Correlation between frequency and G′ and G′′ of 20% GelMA. A Student's *t*-test was conducted to determine the significance; $*p < 0.05$, $**p < 0.01$, $***p < 0.001$, $****p < 0.0001$.

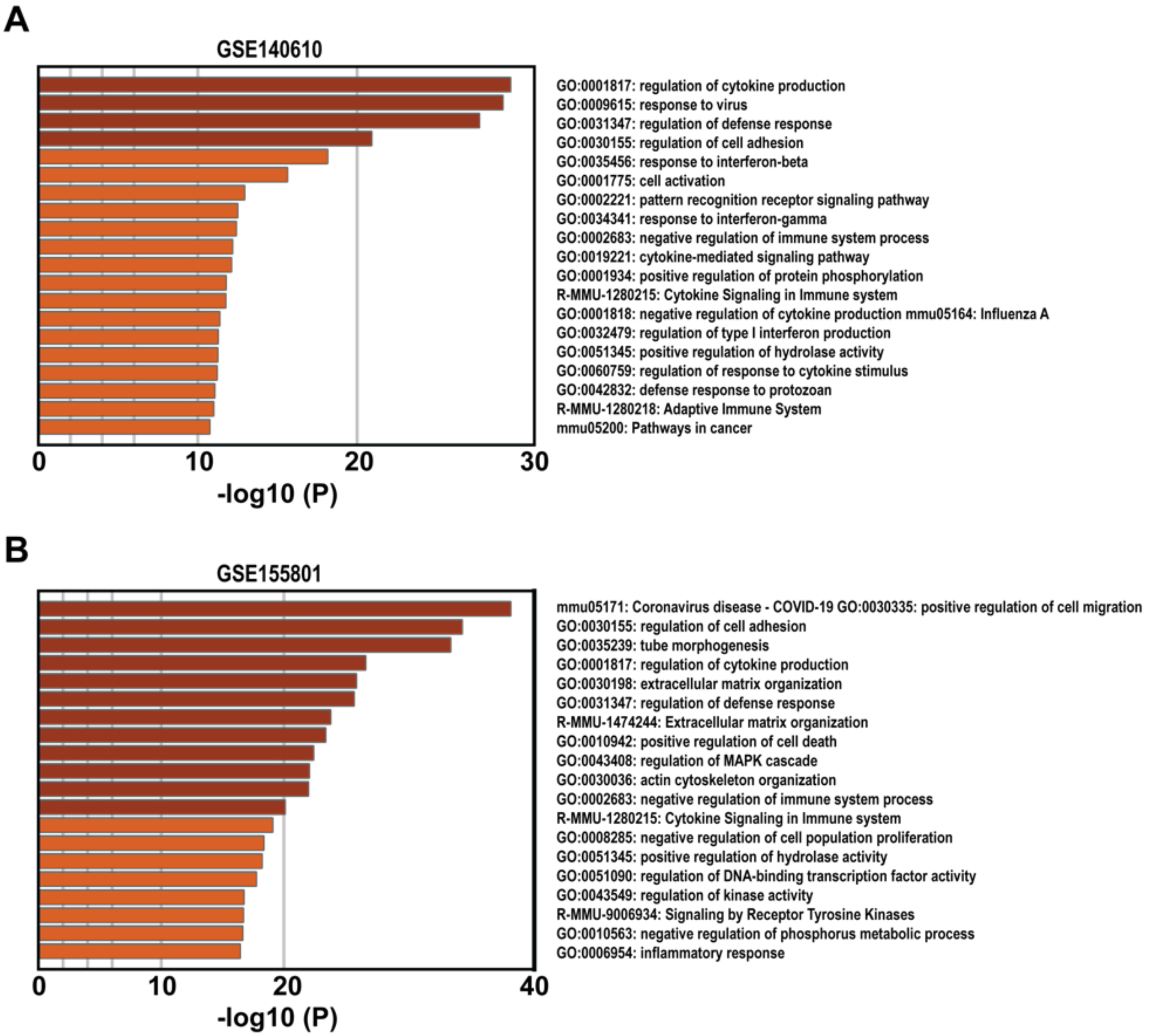

Figure S2. Bar graph of enriched GO terms. (A) Enriched GO terms in GSE140610 datasets. (B) Enriched GO terms in GSE155801 datasets. All statistically enriched terms are selected. The *p*-values and enrichment factors were used as filtering settings. Kappa-statistical similarities were used as a basis for hierarchically clustering other significant terms. In this figure, the threshold of the kappa score was 0.3 for clustering. The bar graphs were produced using Metascape.

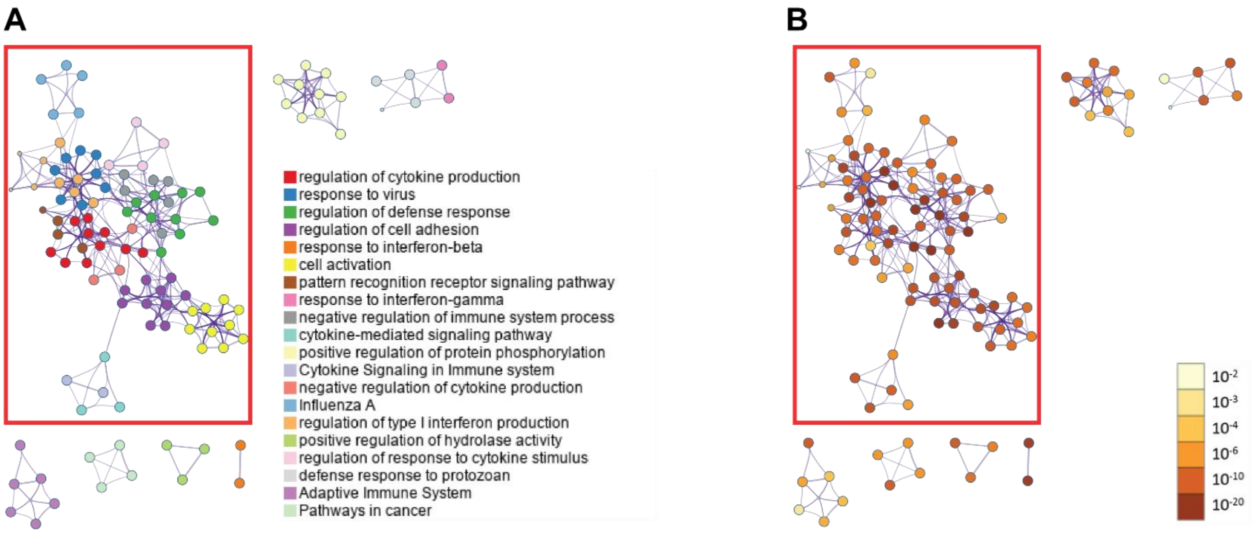

Figure S3. Network of enriched clusters in GSE140610 datasets. The representative GO terms were taken from the whole clusters and transformed into an enrichment network. (A) Network coloured by GO cluster identity. (B) Network coloured by *p*-value; the brightness of a node represents its level of statistical significance (with the legend indicating the *p*-value ranges). The red frame highlights the associated immune clusters.

Note. Each circle node in this network represents one GO term and is coloured by its cluster. Nodes of the same cluster are close to one another. A similarity score of 0.3 was set as a threshold for connecting nodes to each other with a grey line. A thicker line represents a higher similarity score. The network was visualised using Metascape, based on Cytoscape software.

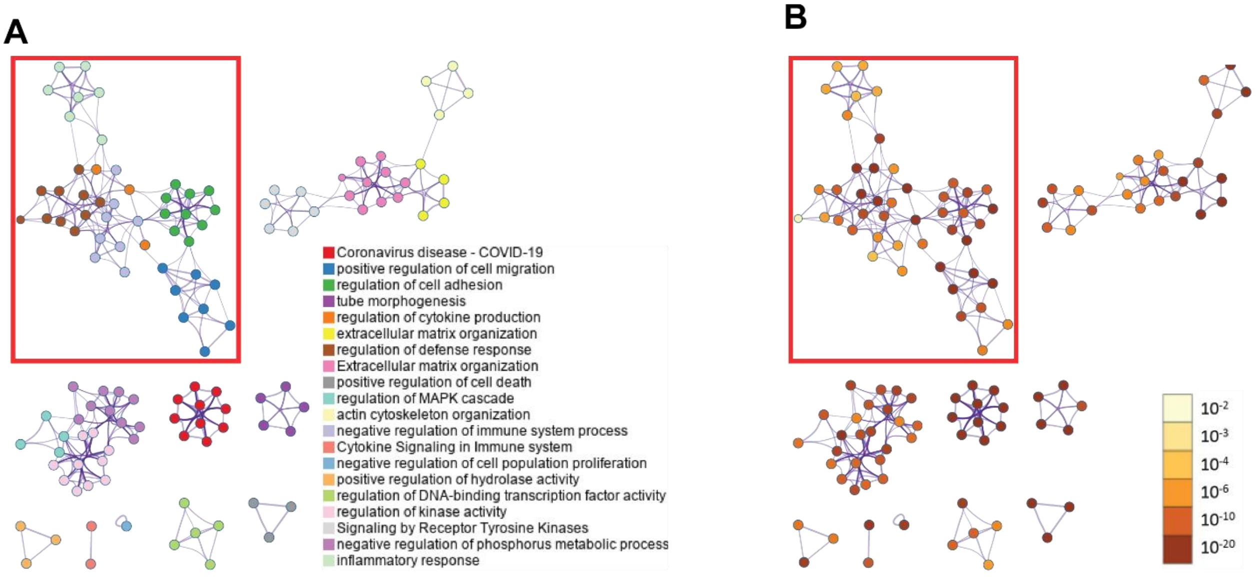

GSE155801

Figure S4. Network of enriched clusters in GSE155801 datasets. The representative GO terms were taken from the whole clusters and transformed into an enrichment network. (A) Network coloured by GO cluster identity. (B) Network coloured by *p*-value; the brightness of a node represents its level of statistical significance (with the legend indicating the *p*-value ranges). The red frame highlights the associated immune clusters.

Note. Each circle node in this network represents one GO term and is coloured by its cluster. Nodes of the same cluster are close to one another. A similarity score of 0.3 was set as a threshold for connecting nodes to each other with a grey line. A thicker line represents a higher similarity score. The network was visualised using Metascape, based on Cytoscape software.

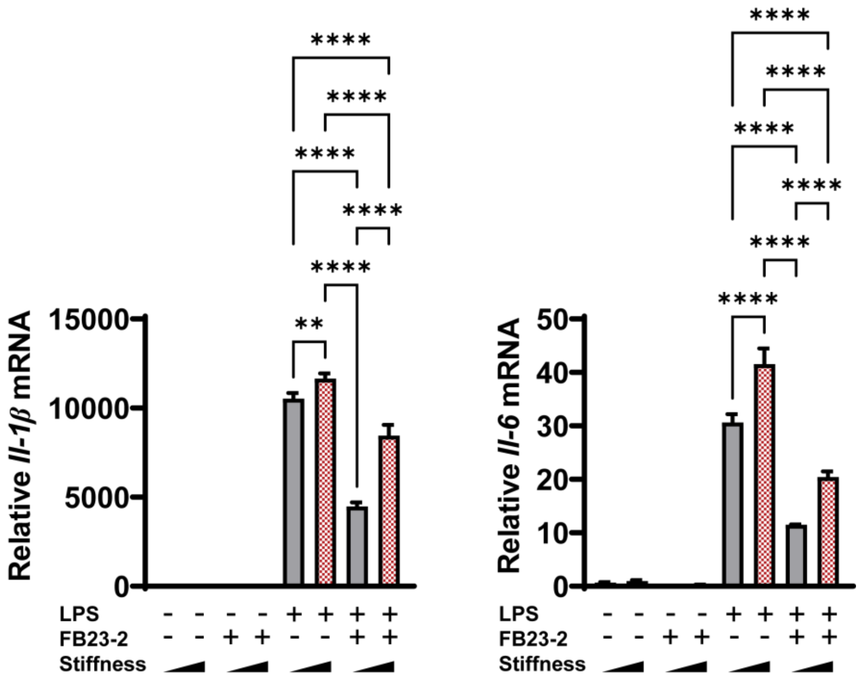

Figure S5. RT-qPCR quantitation illustrating the effect of FB23-2 on the expression of pro-inflammatory genes (Il-1β and Il-6) in BMDMs macrophages on soft or stiff hydrogel with/without LPS stimulation. *$p < 0.05$, **$p < 0.01$; ***$p < 0.001$; ****$p < 0.0001$ by one-way ANOVA with post-hoc multiple comparisons (Tukey's HSD test).

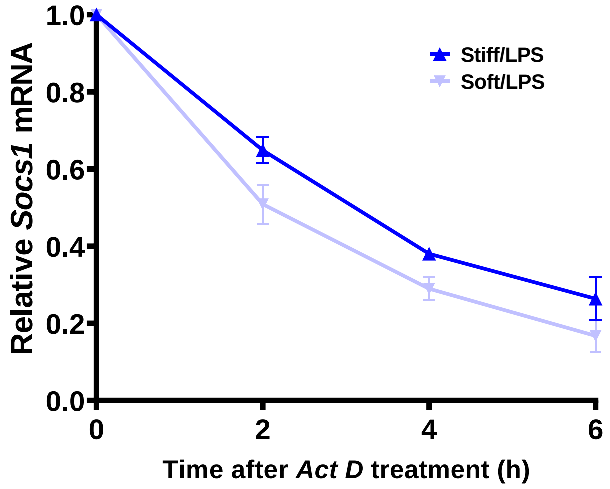

Figure S6. *Socs1* mRNA decaying in macrophages in a stiff or soft microenvironment.

Uncropped Image For Figure 1

The PVDF memberanes were cut in this way.

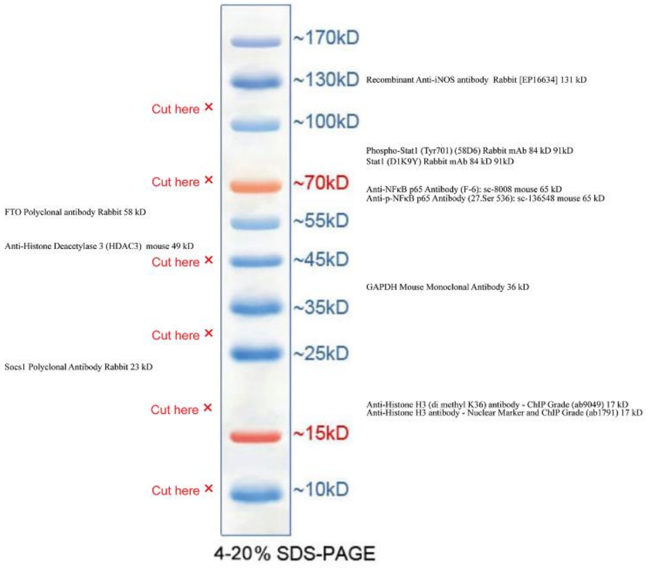

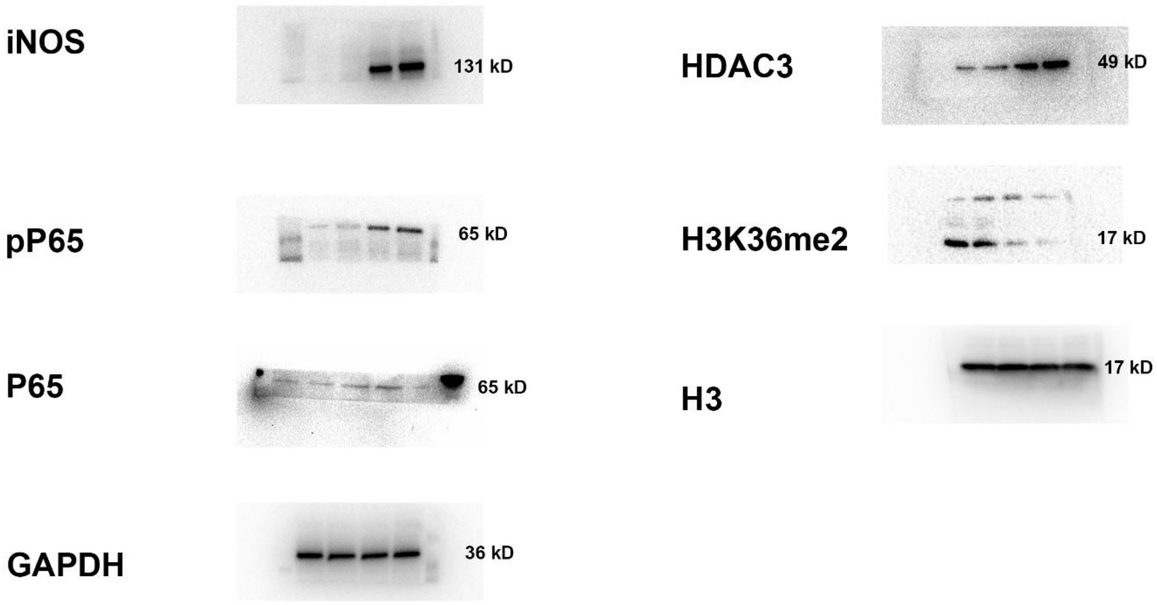

Uncropped Image For Figure 2

SOCS1

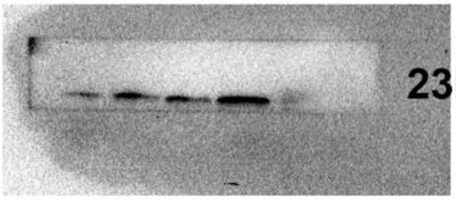

23kD

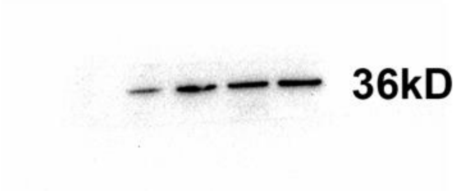

36kD

GAPDH

Uncropped Image For Figure 3

iNOS

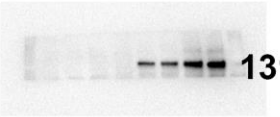

131kD

pp65

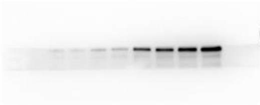

65 kD

pSTAT1

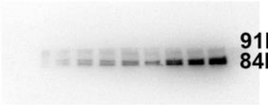

91kD
84kD

p65

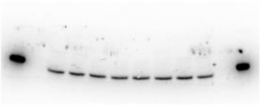

65kD

STAT1

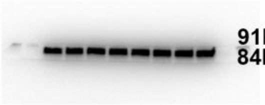

91kD
84kD

GAPDH

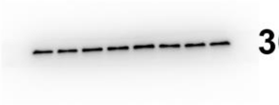

36kD

Uncropped Image For Figure 4

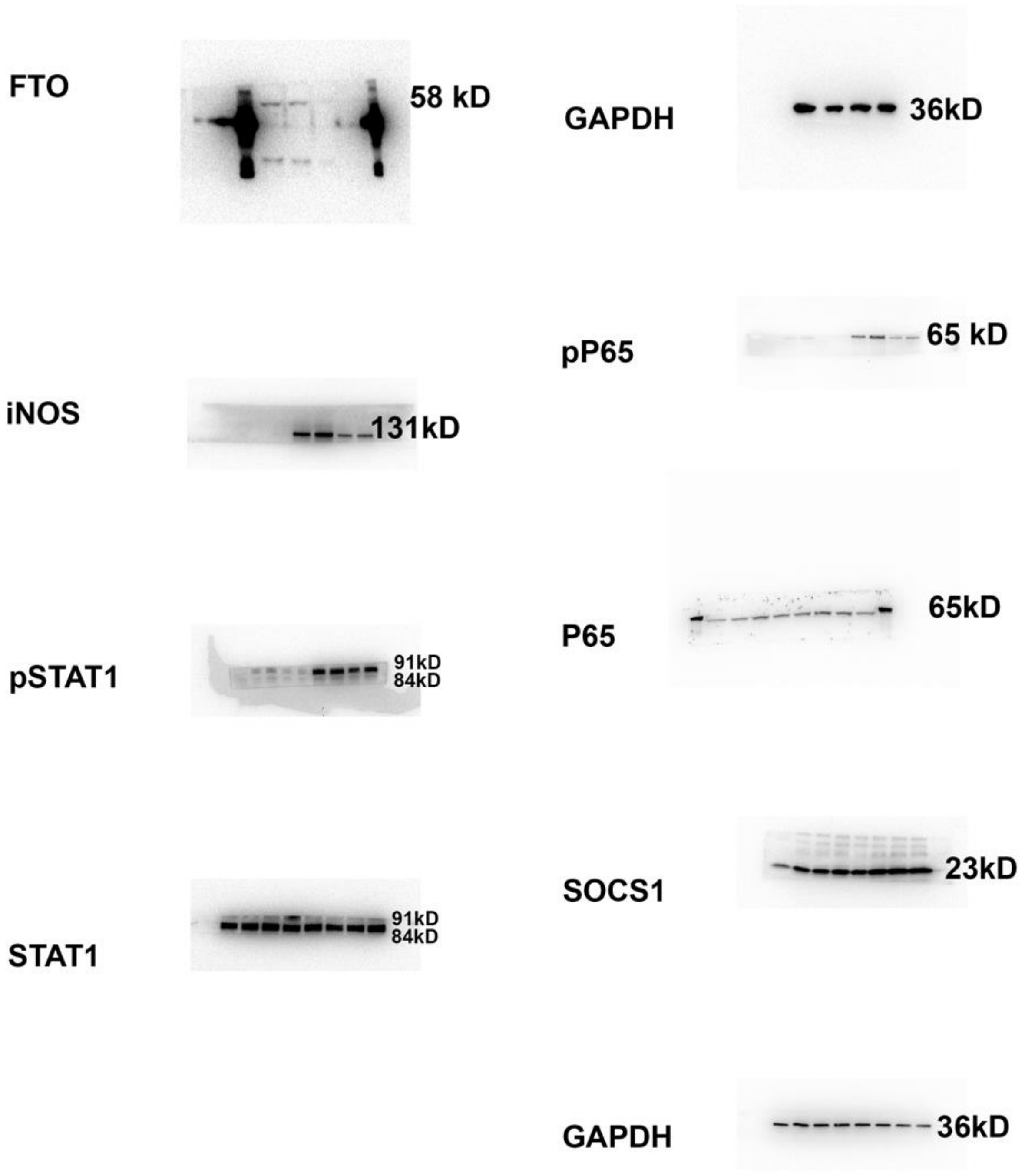

Uncropped Image For Figure 5

FTO

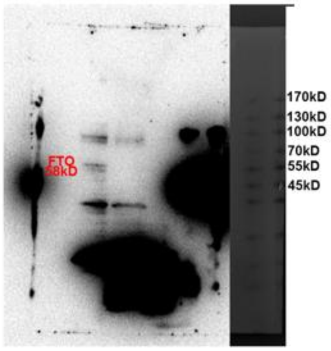

iNOS

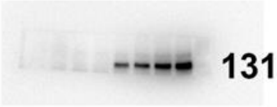

131kD

pSTAT1

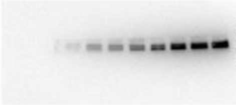

91kD
84kD

STAT1

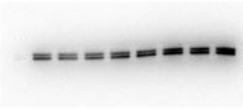

91kD
84kD

GAPDH

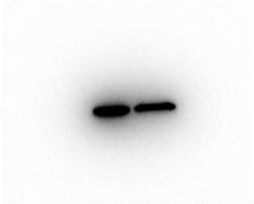

36kD

pP65

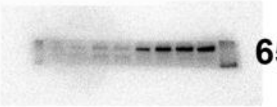

65 kD

P65

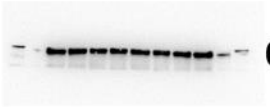

65 kD

SOCS1

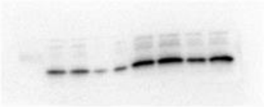

23kD

GAPDH

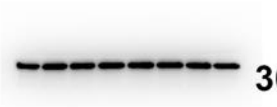

36kD

Uncropped Image For Figure 6

HDAC3 49 kD

H3K36me2 17 kD

H3 17 kD

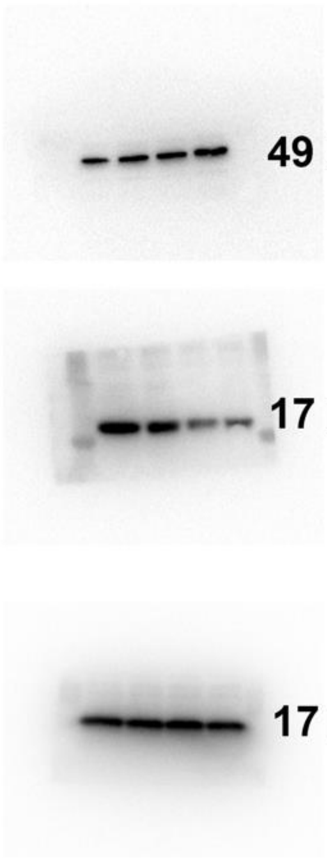

Uncropped Image For Figure 7

SOCS1

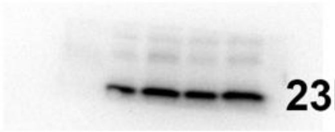

23kD

GAPDH

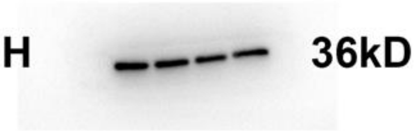

36kD

Table S1. Nucleotide sequence of Primers

qPCR Primers		
**Gene Symbol**	**Sequence**	**Source&Citation**
m-Alkbh5-F	CGCGGTCATCAACGACTACC	PrimerBank ID:31044423a1 ref.42
m-Alkbh5-R	ATGGGCTTGAACTGGAACTTG	PrimerBank ID:31044423a1 ref.42
m-COX2(PTGS2)-F	TGAGCAACTATTCCAAACCAGC	PrimerBank ID:31981525a1 ref.42
m-COX2(PTGS2)-R	GCACGTAGTCTTCGATCACTATC	PrimerBank ID:31981525a1 ref.42
m-FTO-F	TTCATGCTGGATGACCTCAATG	PrimerBank ID:6753916a1 ref.42
m-FTO-R	GCCAACTGACAGCGTTCTAAG	PrimerBank ID:6753916a1 ref.42
m-GAPDH-F	AGGTCGGTGTGAACGGATTTG	PrimerBank ID:6679937a1 ref.42
m-GAPDH-R	TGTAGACCATGTAGTTGAGGTCA	PrimerBank ID:6679937a1 ref.42
m-IL1B-F	GCAACTGTTCCTGAACTCAACT	PrimerBank ID:6680415a1 ref.42
m-IL1B-R	ATCTTTTGGGGTCCGTCAACT	PrimerBank ID:6680415a1 ref.42
m-Il6_F	CCAGGTAGCTATGGTACTCCA	ref.43
m-Il6_R	GCTACCAAACTGGCTATAATC	ref.43
m-METTL14-F	CTGAGAGTGCGGATAGCATTG	PrimerBank ID:30354675a1 ref.42
m-METTL14-R	GAGCAGATGTATCATAGGAAGCC	PrimerBank ID:30354675a1 ref.42
m-METTL3-F	CTGGGCACTTGGATTTAAGGAA	PrimerBank ID:26351497a1 ref.42
m-METTL3-R	TGAGAGGTGGTGTAGCAACTT	PrimerBank ID:26351497a1 ref.42
m-NOS2-F	GTTCTCAGCCCAACAATACAAGA	PrimerBank ID:6754872a1 ref.42
m-NOS2-R	GTGGACGGGTCGATGTCAC	PrimerBank ID:6754872a1 ref.42
m-Socs1-F	CTGCGGCTTCTATTGGGGAC	PrimerBank ID:6753424a1 ref.42
m-Socs1-R	AAAAGGCAGTCGAAGGTCTCG	PrimerBank ID:6753424a1 ref.42
m-TNF-F	CCCTCACACTCAGATCATCTTCT	PrimerBank ID:7305585a1 ref.42
m-TNF-R	GCTACGACGTGGGCTACAG	PrimerBank ID:7305585a1 ref.42
m-WTAP-F	GAACCTCTTCCTAAAAAGGTCCG	PrimerBank ID:33946271a1 ref.42
m-WTAP-R	TTAACTCATCCCGTGCCATAAC	PrimerBank ID:33946271a1 ref.42
m-YTHDF1-F	ACAGTTACCCCTCGATGAGTG	PrimerBank ID:30424609a1 ref.42
m-YTHDF1-R	GGTAGTGAGATACGGGATGGGA	PrimerBank ID:30424609a1 ref.42
m-YTHDF2-F	GAGCAGAGACCAAAAGGTCAAG	PrimerBank ID:225543109c1 ref.42
m-YTHDF2-R	CTGTGGGCTCAAGTAAGGTTC	PrimerBank ID:225543109c2 ref.42
m-YTHDF3-F	CATAGGGCAACAGAGGAAACAG	PrimerBank ID:225543494c1 ref.42
m-YTHDF3-R	ATCTCCAGCCGTGGACCAT	PrimerBank ID:225543494c1 ref.42

ChIP Primers		
**Gene Symbol**	**Sequence**	**Source&Citation**
Socs1_ChIP_Forward	TCC AAG AAG GGT CGA GAT TG	ref.44
Socs1_ChIP_reversed	CCC GCT CTT TTG CTC TAC CT	ref.44
Il6 promoter_Forward	ATGCTCAAGTGCTGAGTCAC	ref.45
Il6 promoter_Reversed	GATTGCACAATGTGACGTCG	ref.45

nos2 promoter_Forward	TCAAGCACACAGACTAGGAG	ref.45
nos2 promoter_Reversed	CCAGAGTCTCAGTCTTCAAC	ref.45
TNF Promoter Forward	TTTCCGAGGGTTGAATGAGA	ref.46
TNF Promoter Reversed	CTGGTTGGCTGCTTGCTTTT	ref.46

RIP/MeRIP primers

Gene Symbol	Sequence	Source&Citation
Socs1 RIP F	GCTGCGGCACAGCGG	ref.20
Socs1 RIP R	CTGTGTCGCCAGCGCAT	ref.20

Table S2. Antibodies

Antibody	SOURCE	IDENTIFIER	Dilution
Anti-Histone H3 (di methyl K36) antibody - ChIP Grade (ab9049)	Abcam	ab9049	WB: 1:1,000; IF1:200
Anti-Histone H3 antibody - Nuclear Marker and ChIP Grade (ab1791)	Abcam	ab1791	WB: 1:1,000
Goat Anti-Mouse IgG H&L (Alexa Fluor® 647) (ab150115)	Abcam	ab150115	IF1:200
Recombinant Anti-iNOS antibody [EP16634]	Abcam	ab202417	WB: 1:1,000
Mounting Medium With DAPI - Aqueous, Fluoroshield (ab104139)	Abcam	ab104139	According to manuscript
Goat Anti-Rabbit IgG H&L (Alexa Fluor 647)	Beyotime	A0468	IF1:200
GAPDH Mouse Monoclonal Antibody	Beyotime	AF0006	WB: 1:1,000
mouse IgG	Beyotime	A7028	ChIP or RIP: 1:30
Phospho-Stat1 (Tyr701) (58D6) Rabbit mAb	Cell Signaling Technology	9167	WB: 1:1,000; IF1:200
Stat1 (D1K9Y) Rabbit mAb	Cell Signaling Technology	14994	WB: 1:1,000
Alexa Fluor(R) 488 Phalloidin, 300 assays	Cell Signaling Technology	8878S	According to manuscript
Anti-mouse IgG, HRP-linked Antibody	Cell Signaling Technology	7076S	WB: 1:3,000
Anti-rabbit IgG, HRP-linked Antibody	Cell Signaling Technology	7074S	WB: 1:3,000
m6A Antibody	Epigentek	A-1801	ChIP or RIP: 1:30
Non-Immune Rabbit IgG	Epigentek	P-9018-24-IgG	ChIP or RIP: 1:30
FTO Polyclonal antibody	Proteintech	27226-1-AP	WB: 1:1,000
YTHDF1 Polyclonal antibody	Proteintech	17479-1-AP	WB: 1:1,000; ChIP or RIP: 1:30
YTHDF2 Polyclonal antibody	Proteintech	24744-1-AP	WB: 1:1,000; ChIP or RIP: 1:30
YTHDF3-specific Rabbit Polyclonal Antibody,150ul	Proteintech	25537-1-AP	WB: 1:1,000; ChIP or RIP: 1:30
Anti-Histone Deacetylase 3 (HDAC3) Antibody (40) | SCBT	Santa Cruz Biotechnology	sc-136290	WB: 1:1,000; IF1:200
Anti-NFκB p65 Antibody (F-6): sc-8008	Santa Cruz Biotechnology	Sc-8008	WB: 1:1,000
p-NFκB p65 (27.Ser 536), 200	Santa Cruz Biotechnology	sc-136548	WB: 1:1,000
Socs1 Polyclonal Antibody	ThermoFisher	PA5-27239	WB: 1:1,000; IF1:200

Table S3. Chemicals

REAGENT or RESOURCE	SOURCE	IDENTIFIER
Western Blot Stripping Buffer	Abcam	ab270550
BeyoMag™ Protein A+G beads	Beyotime	P2108- 1ml
0.5M DTT (DNase, RNase & Protease free)	Beyotime	ST041- 2ml
BeyoColor Multicolor Protein Ladder 10-170 kD	Beyotime	P0076
Recombinant IFN-γ	Beyotime	P5887-10μg
4–20% Mini-PROTEAN® TGX™ Precast Protein Gels	Bio-Rad	4561095
4x Laemmli Sample Buffer	Bio-Rad	#1610747
Immunofluorescence Antibody Dilution Buffer, 100ml	Cell Signaling Technology	12378S
Immunofluorescence Blocking Buffer, 50ml	Cell Signaling Technology	12411S
Opti-MEM® Medium	Gibco	31985070
Silencer™ Select Negative Control No. 1 siRNA	Invitrogen	4390843
LPS-B5 (LPS from E. coli 055:B5) (Standard)	InvivoGen	tlrl-b5lps
YTHDF1 siRNA (m), 10μM	Santa Cruz Biotechnology	sc-155423
SOCS-1 siRNA (m), 10μM	Santa Cruz Biotechnology	sc-40997
FB23-2	Selleck Chem	No.S8837
Recombinant Mouse M-CSF Protein	R&D Systems, Inc	416-ML-050
Actinomycin D	Sigma Aldrich	A1410
Gelatin from porcine skin	Sigma Aldrich	G1890-1KG
Methacrylic anhydride	Sigma Aldrich	276685
TB Green® Premix Ex Taq™ (Tli RNase H Plus)	Takara Bio	RR420A
dNTP Mix (10 mM each)	ThermoFisher	R0192
Halt™ Protease and Phosphatase Inhibitor Cocktail (100X)	ThermoFisher	78440
NuPAGE™ LDS Sample Buffer (4X)	ThermoFisher	NP0007
Oligo(dT)12-18 Primer	ThermoFisher	18418012
Pierce™ Fast Blocking Buffer	ThermoFisher	37575
RIPA Lysis and Extraction Buffer	ThermoFisher	89901
RNaseOUT™ Recombinant Ribonuclease Inhibitor	ThermoFisher	10777019
SuperScript™ III Reverse Transcriptase	ThermoFisher	18080093
Random Primer	ThermoFisher	48190011
Opti-MEM™ I Reduced Serum Medium, no phenol red	ThermoFisher	11058021
Lipofectamine® RNAiMAX Reagent	ThermoFisher	13778150
WesternBright Quantum kit	WesternBright	K-12042
WesternBright Sirius Chemiluminescent Detection Kit	WesternBright	K-12043

Table S4. Commercial Assays

REAGENT or RESOURCE	SOURCE	IDENTIFIER
ChromaFlash High-Sensitivity ChIP Kit	Epigentek	P-2027-48
EpiQuik m6A RNA Methylation Quantification Kit (Colorimetric)	Epigentek	P-9005-96
EpiQuik CUT&RUN m6A RNA Enrichment (MeRIP) Kit	Epigentek	P-9018-24
QIAamp DNA Kits	QIAGEN	56304
QIAshredder	QIAGEN	79656
RNeasy Mini	QIAGEN	74004
Pierce™ BCA Protein Assay Kit	ThermoFisher	23227
TRIZOL PLUS PURIFICATION KIT	ThermoFisher	12183555

Table S5. sgRNA for CRISPR

Target gene	Sequence	PAM	On Target score	Off Target score	Overall score
Fto	CATGAAGCGCGTCCAGACCG	CGG	69.22	1	59
